# Ancient gene duplications have shaped developmental stage-specific expression in *Pristionchus pacificus*

**DOI:** 10.1186/s12862-015-0466-2

**Published:** 2015-09-15

**Authors:** Praveen Baskaran, Christian Rödelsperger, Neel Prabh, Vahan Serobyan, Gabriel V. Markov, Antje Hirsekorn, Christoph Dieterich

**Affiliations:** Max-Planck Institute for Developmental Biology, Spemannstr. 35, Tübingen, 72076 Germany; Max-Delbrück Center for Molecular Medicine, Robert-Rössle-Straße 10, Berlin-Buch, 13125 Germany; Max-Planck Institute for Biology of Aging, Joseph-Stelzmann-Str. 9b, Köln, 50866 Germany

## Abstract

**Background:**

The development of multicellular organisms is accompanied by gene expression changes in differentiating cells. Profiling stage-specific expression during development may reveal important insights into gene sets that contributed to the morphological diversity across the animal kingdom.

**Results:**

We sequenced RNA-seq libraries throughout a developmental timecourse of the nematode *Pristionchus pacificus*. The transcriptomes reflect early larval stages, adult worms including late larvae, and growth-arrested dauer larvae and allowed the identification of developmentally regulated gene clusters. Our data reveals similar trends as previous transcriptome profiling of dauer worms and represents the first expression data for early larvae in *P. pacificus*. Gene expression clusters characterizing early larval stages show most significant enrichments of chaperones, while collagens are most significantly enriched in transcriptomes of late larvae and adult worms. By combining expression data with phylogenetic analysis, we found that developmentally regulated genes are found in paralogous clusters that have arisen through lineage-specific duplications after the split from the *Caenorhabditis elegans* branch.

**Conclusions:**

We propose that gene duplications of developmentally regulated genes represent a plausible evolutionary mechanism to increase the dosage of stage-specific expression. Consequently, this may contribute to the substantial divergence in expression profiles that has been observed across larger evolutionary time scales.

**Electronic supplementary material:**

The online version of this article (doi:10.1186/s12862-015-0466-2) contains supplementary material, which is available to authorized users.

## Background

The development from a single cell progenitor to a fully grown multicellular organism with up to hundreds of tissue types is accompanied by differentiation processes that change the transcriptional state, i.e. the levels of gene expression within each cell type. The nematode *Caenorhabditis elegans* has been proven as a highly attractive model for studying developmental biology, as its ontogenesis resembles an almost completely deterministic process that results in a fixed number of cells in the mature animal. In addition, cell ablation experiments constitute a powerful tool to study development upon perturbation [1, 2]. Over the last two decades, the nematode *Pristionchus pacificus* has been established as a satellite model organism to *C. elegans*. Both nematodes are predominantly selfing species, where most individuals are hermaphrodites with the potential of self-fertilization. Only a small fraction of offspring will be males that can mate with hermaphrodites and thus cause recombination between different lineages. Additionally, both nematodes develop through four larval stages and can enter a growth-arrested dauer stage under harsh environmental conditions [3, 4]. Phylogenetic analyses have grouped *C. elegans* and *P. pacificus* into the same clade [5] but nevertheless revealed substantial sequence divergence, i.e. *P. pacificus* has a roughly five times higher protein sequence divergence to *C. elegans* than another member of the *Caenorhabditis* genus [6, 7]. Previous comparative studies of development between *C. elegans* and *P. pacificus* have also demonstrated that sequence divergence is correlated with divergence in a number of developmental processes [8–10]. The possibility of genome-wide expression profiling methods such as microarrays and RNA-seq have motivated several studies to characterize the transcriptional changes that guide cell proliferation and differentiation proccesses throughout development [11, 12], and to identify general patterns in the evolution of gene expression [12–15].

For *C. elegans* and *P. pacificus*, the same features that make them excellent model organisms for studying the genetics of developmental processes, such as their small size and short generation time, also complicate detailed studies of development on a transcriptional level. Thus, relative to vertebrate model systems such as mouse and human cell lines, only a limited number of studies exists that focused on *C. elegans* development at a transcriptional level [11, 12, 14, 16]. Moreover, there is only a single gene expression profiling study of different developmental stages in *P. pacificus* [9]. This study focused on the comparison between the growth arrested dauer stage and worms that exited from the dauer stage [9]. Sinha *et al.* showed that, despite the fact that some key actors initiating the signaling cascades into dauer entry are conserved between *C. elegans* and *P. pacificus* [4], dauer specific transcriptome profiles show substantial divergence with only 184 genes that showed dauer-specific differential regulation in both species, of which 68 genes even exhibited opposing trends with respect to up and downregulation [9].

In this study, we present the first gene-expression profiling data for early larval stages of *P. pacificus*. In total, we have sequenced ten RNA libraries that were collected throughout larval development of *P. pacificus*. We identify and characterize gene clusters with developmental-stage specific expression and use phylogenetic analyses to test for conservation of stage-specific expressed genes. Our findings suggest that a large portion of genes that are expressed in a developmental-stage specific manner have arisen by ancient duplication events within the *Pristionchus* lineage.

## Methods

### Staging

Culturing of worms was carried out on NGM agar plates seeded with 150 mg *E. coli* OP50 pellet obtained from liquid culture in LB medium. For harvesting, plates full of embryos and gravid adults were rinsed with M9 buffer. Worms were spun down at 1300 g for 3 min at 4 ^∘^C with low deceleration and the pellet was washed three times with M9. To kill all larvae, the pellet was resuspended in bleach solution [1 % NaClO, 0.5 M NaOH in M9] and incubated for 10 min with periodical vortexing every 2 min. Diluting 1:3 with sterile M9 stopped the bleaching reaction. Worms and eggs were spun down and washed twice. Sucrose flotation was done to obtain pure eggs: The pellet was well resuspended in 6 ml of sterile, cold M9. Holding the tube at an angle, 4 ml of sterile and cold 60 % sucrose solution and another 2 ml of M9 were added, followed by 2 min of centrifugation at 485 g. The upper, whitish layer was transferred to a fresh conical tube and washed twice with sterile M9. The purified eggs were brought up in 30 ml of sterile M9 and left to hatch overnight, shaking at 200 rpm and 20 ^∘^C. With the help of a binocular, hatched larvae were counted in 1 *μl* aliquots dripped on a glass slide. To obtain juvenile stages J2, J3 and J4 as well as the adult stage about 80,000 larvae were washed with sterile M9 and equally distributed to four 15 cm NGM agar plates seeded with OP50. After 10 h of incubation at 20 ^∘^C for J2, 30 h for J3, 44 h for J4 and 56 h for adults, worms were rinsed off in a 15 cm plate with sterile and cold M9, collected in a conical tube and washed three times. The final worm pellet was set on ice for immediate RNA isolation. To obtain dauer larvae about 200, 000 worms were incubated for four days in a 100 ml flask containing 5 ml of sterile M9 and 50 *μg* OP50 (= 40 larvae /*μl* and 10 mg OP50/ml), shaking at 200 rpm and 20 ^∘^C. Dauer culture was diluted with sterile M9 and spun down for 5 min at 1650 g and 4 ^∘^C with low deceleration. To kill all non-dauer larvae, the pellet was resuspended in 50 ml of 1 % SDS solution and left on a tube roller for 15 min. After centrifugation the pellet was washed three times with sterile M9. Sucrose flotation was done to purify dauer culture: The washed pellet was well resuspended in 14 ml of sterile and cold 30 % sucrose solution (in M9) in a 15 ml conical tube, spun at 50 g for 1 min and immediately accelerated to 1150 g for another 3 min. Larvae from the upper and interphase were transferred to a new tube and washed three times with sterile M9. Purified dauers were brought up in 30 ml of sterile M9 and left to recover overnight, shaking at 200 rpm and 20 ^∘^C. Dauer larvae were spun down and the pellet was set on ice for immediate RNA isolation. For all stages (J2, J3, J4, adult, and dauer), we generated two independent worm cultures (biological replicates).

### RNA isolation and library preparation

Each worm pellet was resuspended in 10 volumes of Trizol. 1 ml of Trizol-worm mixture was transferred to a fresh screw-cap vial filled to 500 *μl* with glass beads and homogenized in a Precellys homogenisator for 2 ×10 sec, 6000 rpm, 5 sec pausing. The homogenate was transferred to a RNase-free tube and left to incubate for 10 min at room temperature. RNA was isolated by a single chloroform extraction and precipitation with isopropanol for 30 min at −20 ^∘^C. The pellet was washed with 80 % ethanol, dried and resuspended in 50 *μl* of RNase-free water. To determine RNA concentration and quality Qubit and Bioanalyzer measurements were performed. Only samples with RINs ≥ 9 were used for subsequent library preparation. Libraries were obtained from 2 *μg* RNA. Multiplexed single-end sequencing of 50 nt reads was performed on an Illumina HiSeq machine with all 10 libraries pooled on one lane of the sequencing flow cell.

### RNA-seq analysis

Raw reads were aligned to the *P. pacificus* Hybrid1 genome assembly using tophat (version 2.0.3) [17]. Cufflinks (version 2.0.1) was used to quantify expression levels as FPKM values in all ten libraries for all predicted genes [17]. We used the ‘prcomp’ function of the R package ‘stats’, to perform a PCA for all genes with Cufflinks FPKM values >0. In parallel, we used the program Cuffdiff (version 2.0.1) in blind mode, i.e. without information about biological replicates, to predict significant differential expression across all pairwise comparisons (FDR <0.01). For the Cuffdiff results, we used a hierarchical clustering approach, implemented in R, to complement the results obtained from the PCA.

To identify clusters of coregulated genes, we applied the biclustering algorithm SAMBA [18], implemented in the Expander package (version 6.3.1), to the pairwise results from Cuffdiff and run in blind mode. We generated an *n*×*m* matrix with *n* genes and *m*=45 comparisons, with the individual entries indicating whether a gene was significantly differentially expressed (-1 : = downregulation and 1: = upregulation) or not. We then loaded the matrix as relative expression data into Expander and ran SAMBA using default settings, resulting in 29 biclusters.

FPKM values for all genes (Cufflinks), fold changes and FDR corrected p-values (Cuffdiff) for all genes that were found to be significantly differentially expressed in at least one comparison, and the assignments of genes to expression biclusters (SAMBA) are shown in Additional file 1.

### Validation by qRT-PCR

In order to confirm our classification of transcriptomes into early larvae, dauer larvae, and late larvae including adults, we obtained clean J2 and dauer cultures using recently developed protocols described in Bose et al. [19] and Penkov et al. [20] and measured expression levels for six candidate genes by qRT-PCR. Adult worms were obtained by manually picking. qRT-PCR experiments were performed as described previously [21] using *Ppa-cdc-42* and *Ppa-y45F10D.4* as reference genes.

### Protein domain annotation and definition of orthology relationships

We used the program hmmsearch (option -E 0.001) to search for known protein domains in the set of 30,884 predicted *P. pacificus* protein sequences (version TAU [9]), as defined by the PFAM database. The search program hmmsearch and the PFAM domain database were both obtained from the HMMER package (version 3.0).

In order to identify homologs for *P. pacificus* genes, we downloaded protein sequences for *C. elegans*, *C. briggsae*, *C. angaria*, *Haemonchus contortus*, *Meloidogyne hapla*, *Brugia malayi*, *Bursaphelenchelus xylophilus*, *Ascaris suum*, and *Trichinella spiralis* from Wormbase WS230 and *Heterorhabditis bacteriophora* sequences from Wormbase WS231. Furthermore we downloaded protein sequences for *Loa loa* and *Wuchereria bancrofti* from the filarial worms sequencing project, Broad Institute of Harvard and MIT (http://www.broadinstitute.org), *Meloidogyne incognita* protein sequences from the *M. incognita* resources website (http://www6.inra.fr/meloidogyne_incognita), *Panagrellus redivivus* sequences from a website provided by Jagan Srinivasan, and protein sequences for *Dirofilaria immitis* from nematodes.org. Homologs for *P. pacificus* were identified by searching these data sets for BLASTP (version 2.2.28+) hits with e-value <0.001. This resulted in 20,999 (68 %) *P. pacificus* proteins with homologs in other nematode species and 9885 (32 %) *P. pacificus* proteins without homologs (orphan genes).

We predicted one-to-one pairs between *P. pacificus* and *C. elegans* by using a variant of the widely employed methodology of best-reciprocal hits [6, 9, 22]. More precisely, we first defined inparalogs and then assigned best-reciprocal hits as one-to-one orthologs, only if neither the *C. elegans* nor the *P. pacificus* protein had any inparalog. Hereby, inparalogs were defined by following a similar methodology as implemented in the Inparanoid method [23], i.e. by identifying intraspecies BLASTP pairs that are more closely related than the best inter-species pairs. This procedure predicted 5985 one-to-one orthologous pairs. We evaluated the quality of one-to-one orthology predictions using a data set of 107 *C. elegans* genes for which orthology relationships were manually investigated using alternative versions of *P. pacificus* gene predictions, TBLASTN searches to complement incomplete gene models, and subsequent phylogenetic analysis including all potential paralogous sequences. Out of 57 *C. elegans* genes with manually identified one-to-one orthologs 42 were also correctly predicted by our automatic method. For 50 *C. elegans* genes, for which manual analysis could not identify one-to-one orthologs, 48 did not have predicted one-to-one orthologs by our automatic method. False and missing orthology assignments could be attributed in most cases to missing or only incomplete gene predictions. Although our set of manually annotated *C. elegans* genes is not a random subset and therefore is not representative for the whole genome, our results indicate that even though a potentially large fraction of true orthology relationships were missed, most of the predicted one-to-one orthologs are indeed correct. The evaluation results and the manually curated sequences for the *P. pacificus* orthologs of 57 *C. elegans* are presented in the Tables S4 and S5 and Data S1 (Additional file 2).

The set of *P. pacificus* orphan genes and genes with homologs in other nematode species (excluding *P. pacificus* one-to-one orthologs with *C. elegans*), were further subdivided into singleton sequences and genes with putative paralogs by computing an adjacency matrix out of BLASTP hits within *P. pacificus* and extracting all connected components. Proteins that were members of connected components of size greater than one, were classified as “with paralogs”, or “singletons” otherwise. In the case of genes with homologs in other nematode species, the category “with paralogs” (*N*=11,919) represents multiple *P. pacificus* genes with many-to-many, many-to-one, and many-to-zero orthology relationships [24] with respect to *C. elegans* genes, while singletons (*N*=3095) represent one-to-many orthology relationships. Similarly, the 9885 *P. pacificus* orphan genes were divided into 4820 genes with paralogs and 5065 singletons.

### Phylogenetic analysis

Multiple sequence alignment for HSP20 and HSP70 protein sequences were computed using Clustal Omega tool [25]. In order to test which model of amino acid substitution better explain the evolution of these proteins we have used Prottest server [26]. For both analyzed gene families, the LG substitution model was identified as best model by Prottest. The final maximum likelihood trees (Figs. 3 and 4) were constructed using LG substitution model, as implemented in the R Library Phangorn [27].

## Results

### Distinct transcriptome profiles in early larvae, dauer, and adult worms

**Fig. 1:**
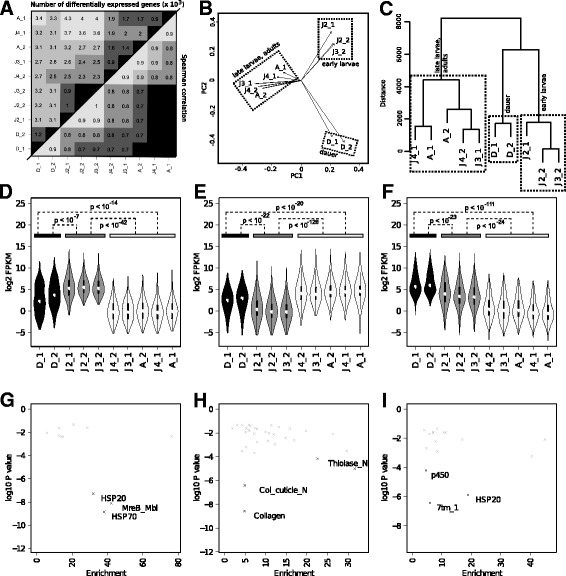
Comparison and clustering of developmental transcriptomes. **a** Correlation of expression values and numbers of significantly differentially expressed genes across all pairwise comparisons. The upper left triangle of the matrix indicates the number of genes (×10^3^) that were predicted to be significantly differentially expressed by Cuffdiff and the lower right triangle shows Spearman correlations of expression values. **b** Principal component analysis of expression values as measured by Cufflinks, indicates that the transcriptomes can be grouped into three clusters. **c** Hierarchical clustering of transcriptomes based on the pairwise comparisons of all samples using Cuffdiff. **d**–**f** Violin plot of expression values in all samples for genes clustered in bicluster 4 (panel **d**), 12 (**e**) and 24 (**f**) respectively. Color code indicates the rough grouping of samples into three developmental stages. The statistical significance of expression differences across stages is shown as the maximum p-value (Wilcoxon test) between any pairwise comparisons of samples across stages. **g**–**i** Enrichment of Pfam domains in bicluster 4 (**g**), 12 (**h**) and 24 (**i**) respectively. The plots show the enrichment factor vs. the significance (−*l* *o* *g*_10_ *P*). The most significant protein families are highlighted in the individual plots

In order to investigate temporal patterns of gene expression in *P. pacificus*, we decided to sequence transcriptome libraries that were sampled throughout the development of *P. pacificus*. The chosen stages comprise larval stages J2, J3, J4, and dauers as well as libraries of adult worms. Between 13 and 17 million reads per library were obtained by sequencing on the Illumina platform. We estimated expression values samples as number of fragments per kilobase transcript per million reads sequenced (FPKM) and quantified variation in expression profiles across all samples by calculating Spearman correlations of FPKM values and by counting the number of genes with significantly different read counts as determined by the software Cuffdiff (Fig. 1a). In general, all profiles show correlation coefficients *ρ*≥0.6 (Spearman) and showed significant differential expression in up to four thousand genes (Fig. 1a). These results suggest that the relative normalization of expression levels relative to the expression of all genes, as commonly applied in analysis of RNA-seq data [17, 28], is indeed valid for the sequenced *P. pacificus* samples and that therefore all data sets are comparable.

Next, we compared expression profiles of all ten samples by performing principal component analysis on the FPKM expression values (Fig. 1b). The first two principal components explain 56 % and 25 % of global variability and indicate that the different samples represent three distinct developmental stages: 1) an early larval stage, 2) dauer larvae, and 3) late larvae and adult worms. While the early stage contains samples that were labeled as J2 and J3, the adult stage is a mix between samples that were labeled as J3, J4, and adult worms. This suggests that our staging protocol resulted in an imperfect synchronization of worm cultures. Consequently, this only allows a rough assignment of samples to developmental stages. We attribute this to the fact that in contrast to *C. elegans*, where larvae hatch during the L1 stage, in *P. pacificus*, the first molt takes place within the egg and hatching takes place during the J2 stage [3]. Thus, bleaching methods, which eliminate all hatched worms, will still retain embryos, J1, and J2 larvae in *P. pacificus*.

The classification of transcriptomes into roughly three developmental stages was further supported by hierarchical clustering based on Euclidean distances that were calculated from a matrix of numbers of genes that were identified as being significantly differentially expressed in pairwise comparisons by Cuffdiff (Fig. 1c). To investigate the substructure in the transcriptomes of late larvae and adult worms, we ordered all transcriptomes using a PCA based approach implemented in the software BLIND [29]. According to this approach, the transcriptomes labeled as J4_1 and A1 were predicted as being from later stages than J3_1, J4_2 and A2.

Finally, we used qRT-PCR experiments of six candidate genes to confirm developmental regulation and to validate the classification of transcriptomes (Fig. 1b,c). We therefore generated clean J2 and dauer cultures, as well as manually picked adults for quantification of expression levels (see *Methods*). Five out of the six candidate genes showed expression levels that are very consistent with the RNA-seq data (Additional file 2: Figure S2), indicating that the measured expression levels are reproducible and that our classification of transcriptomes is correct.

### Clusters of developmentally regulated genes

Given the uncertainty of the exact stages of the sequenced samples that only allows for a rough classification into the three aforementioned clusters: early larvae, late larvae with adults, and dauer larvae, we decided to discard the labeling of samples and to perform a blind analysis of the data in order to identify genes with differential expression across the timecourse. We only use the rough grouping of samples for later interpretation of the identified clusters. To define clusters of developmentally regulated genes, we used an unsupervised biclustering approach, as implemented in the software SAMBA [18]. Based on the data of significant differential expression across all pairwise comparisons, SAMBA tries to identify subsets of genes that show correlated expression profiles in a subset of comparisons. One major rationale to use a biclustering approach is that genes may exhibit correlated expression profiles under certain conditions, but not in other conditions [18]. For example, if a common major regulator of a set of target genes is not expressed at a given stage, the expression of target genes will then be controlled by other factors that are not necessarily shared between all the genes, giving rise to divergent expression patterns at a particular stage. Thus the use of a biclustering approach drops the assumption of a strict correlation in expression patterns across all stages. In total, SAMBA identified 29 partially overlapping biclusters (Fig. 1d–f and Additional file 2: Figure S1) that contain 5161 (17 %) of predicted *P. pacificus* genes. Figure 1d–f show the distribution of expression levels for three exemplary biclusters (Bicluster 4, 12, and 24) across all ten transcriptomes. Each of the three biclusters exhibits highest expression levels at different developmental stages. Figure S1 (Additional file 2) shows the distributions of expression values for all biclusters, all of which exhibit substantial expression variation across the ten samples. We therefore choose to treat all genes, that were identified by the biclustering approach as developmentally regulated genes. To contrast this set with genes that do not show developmental regulation, we defined a set of 5151 house-keeping genes that showed consistently robust expression (FPKM ≥10) in all samples and did not exhibit any significant differential expression in any of the comparisons.

We characterized the resulting gene sets by performing a Gene Ontology (GO) analysis based on *C. elegans* one-to-one orthologs using the David functional annotation webtool [30]. The dauer-specific bicluster 24 was most significantly enriched for G-protein coupled receptor protein signaling pathway (GO:0007186, 10-fold enriched, *P*<10^−15^) and neuropeptide signaling pathway (GO:0007218, 30-fold enriched, *P*<10^−12^). Other biclusters showed strong enrichment in biological processes such as molting cycle (GO:0042303), cell projection organization (GO:0030030), hedgehog receptor activity (GO:0008158), and chitin metabolic process (GO:0006030). In contrast, house-keeping genes only showed a strong overrepresentation of ribosomal proteins (Additional file 2: Table S1).

### Comparison with previous expression-profiling studies

We compared our data set with three previous gene expression profiling studies on *P. pacificus*: a study that compared expression profiles of dauer larvae and worms that have exited dauer stage [9], a research that profiled expression in germline ablated worms which exhibitted a strongly extended lifespan [31], and a study that measured expression differences after exposure to four different pathogens [32]. Table S2 (Additional file 2) shows a summary of all biclusters of developmentally regulated genes, which showed a significant overlap (*P*<0.01) with any of the previously identified gene sets [9, 31, 32]. When compared to the dauer *vs.* dauer exit experiment, five out of the six biclusters that show most significant enrichment with genes upregulated in dauers *vs.* dauer exit worms, also show trends for higher expression in dauers *vs.* adult worms and late larvae (Additional file 2: Figure S1). Similarly, the six biclusters that show most significant enrichment with genes downregulated in dauers *vs.* dauer exit worms, also show trends for lower expression in dauers *vs.* adult worms and late larvae (Additional file 2: Figure S1). Although the data sets are not fully comparable because dauer-exit worms are not equivalent to adult worms, these findings further support that our expression measures based on RNA-seq experiments are largely robust and reproducible when compared to expression data obtained from microarrays [9].

In a previous study, Rae and Sinha et al. found that germline ablations in *P. pacificus* lead to increased longevity [31]. In comparison with germline ablated worms, the most significant association was a four fold enrichment of house-keeping genes in genes that are downregulated upon germline ablation (*P*<10^−300^). We interpret this finding as evidence, that general metabolic processes are slowed down in germline ablated animals. On the contrary, most developmentally regulated clusters were found to be significantly depleted among genes that are downregulated upon germline ablation.

Next, we compared the identified gene sets with the transcriptional response of *P. pacificus* worms to four different pathogens (*Xenorhabdus nematophila, Serratia marcescens*, *Staphylococcus aureus*, *Bacillus thuringiensis*) [32]. Again, the most significant associations, were that house-keeping genes were significantly enriched in genes that are downregulated upon exposure to *Xenorhabdus nematophila* and *Serratia marcescens*. As these two pathogens kill most of *P. pacificus* worms within four days [32], we interpret these overlaps as a result of pathogenicity associated necrosis, which leads to a breakdown of house-keeping functions in dying cells.

### Overrepresentation of gene families in stage-specific expression biclusters

To gain further insight into the putative functions of developmentally regulated genes we characterized these gene sets by testing for overrepresentation of predicted protein domains. Overrepresentation analysis of protein domains represents a complementary approach to the previous GO enrichment analysis since the GO analysis was restricted only to genes with one-to-one orthologs in *C. elegans*, whereas the protein domain prediction was applied on all *P. pacificus* genes (see *Methods*).

The complete results of the protein domain overrepresentation analysis for all biclusters are shown in Table S3 (Additional file 2). As examples, we will now discuss three of them in more detail (Fig. 1g–i). Bicluster 4, which exhibits highest expression at early larval stages, shows strongest enrichments for actin-like MreB proteins (PF06723), and two classes of heat shock proteins HSP20 (PF00011) and HSP70 (PF00012) (Fig. 1f). In contrast, bicluster 12 which shows highest expression in adults including late larvae, is highly significantly overrepresented in thiolase genes (Fig. 1h, PF00108) that are associated with fatty acid metabolism. Interestingly, the dauer-specific bicluster 24, shows also an overrepresentation of HSP20 proteins, suggesting divergent roles of different members of this gene family throughout larval development. In addition, bicluster 24 also shows highly significant enrichments for Cytochrome P450 (PF00067) and G-protein-coupled receptors (PF00001) (Fig. 1i). Studies of *C. elegans* have shown that GPCRs are important for sensing the chemical environment [33]. Given, that in the wild, *P. pacificus* nematodes are assumed to enter dauer stage upon depletion of food sources and start to disperse in order to search for a new host, GPCRs are a plausible candidate gene family to be involved in this process.

### Correlated expression patterns of paralogous clusters in HSP gene families

**Fig. 2:**
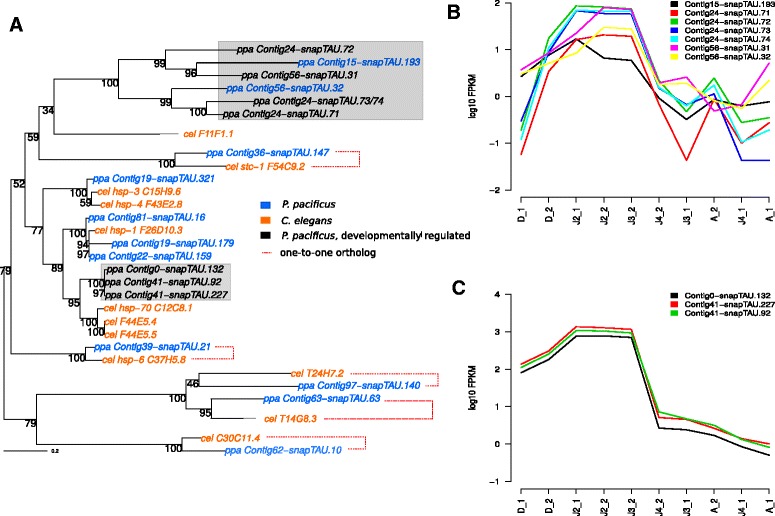
Gene duplications in the HSP70 gene family. **a** Phylogenetic tree of HSP70 genes from *C. elegans* and *P. pacificus*. In *P. pacificus*, a cluster of six paralogous genes that have likely arisen by lineage-specific gene duplications shows high expression in early larvae (**b**), a second cluster of three paralogous genes shows also highest expression levels in early larvae (**c**)

**Fig. 3:**
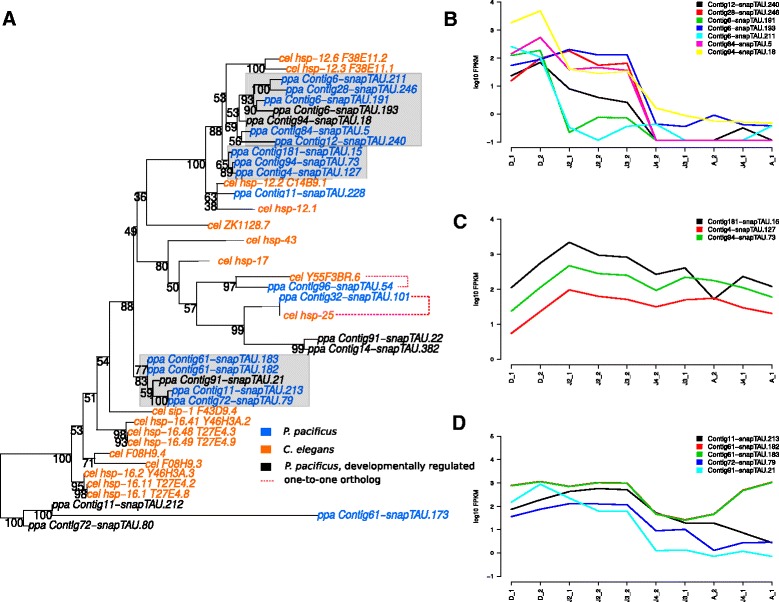
Gene duplications in the HSP20 gene family. **a** Phylogenetic tree of HSP70 genes from *C. elegans* and *P. pacificus*. **b**–**d** Expression profiles for genes in individual paralogous groups suggest correlated expression within subtrees but distinct profiles across subtrees

Previous studies have shown divergent profiles of dauer transcriptomes between *C. elegans* and the remotely related nematodes *P. pacificus* and *Strongyloides stercoralis* [9, 34]. To extend these dauer stage restricted comparisons to a broader developmental time-scale, we tested, whether developmentally regulated genes have one-to-one orthologs in *C. elegans*. For this purpose, we reconstructed phylogenies of *C. elegans* and *P. pacificus* members of the HSP70 and HSP20 gene families (Figs. 2a and 3a) for which individual members showed evidence of developmental regulation (Fig. 1g and i). Interestingly, in both cases, we could not detect any developmentally regulated gene with a one-to-one ortholog in *C. elegans*. In contrast, visual inspection of the the distribution of developmentally regulated genes within the trees, shows a clustering of developmentally regulated genes in *P. pacificus*-specific subtrees. This suggests that they represent paralogs that have arisen by gene duplications in the *Pristionchus* lineage. Hypothesizing that the common ancestor of these paralogs was itself already developmentally regulated, we tested how similar expression profiles in individual subtrees of the HSP families are (Fig. 2b–c, Fig. 3b–d). As expected, expression patterns of paralogs showed strong agreement across the ten transcriptomes, even if a gene was not captured by our biclustering approach due to missing significance in the pairwise differential expression analysis. Interestingly, in the case of the HSP20 family, distinct paralog groups showed different expression profiles, such as the genes shown in Fig. 3b which have highest expression in dauers and the genes in Fig. 3c which exhibit highest expression in early larvae. Even within a single paralog group, we see some evidence for divergent expression profiles (Contig61-snapTAU.182 and Contig61-snapTAU.183 being highly expressed in adults when compared to the other genes in Fig. 3d), however more experimental analysis is needed to allow a more robust investigation of this potential subunctionalization.

### Developmentally regulated gene clusters are enriched for duplicated genes

**Fig. 4:**
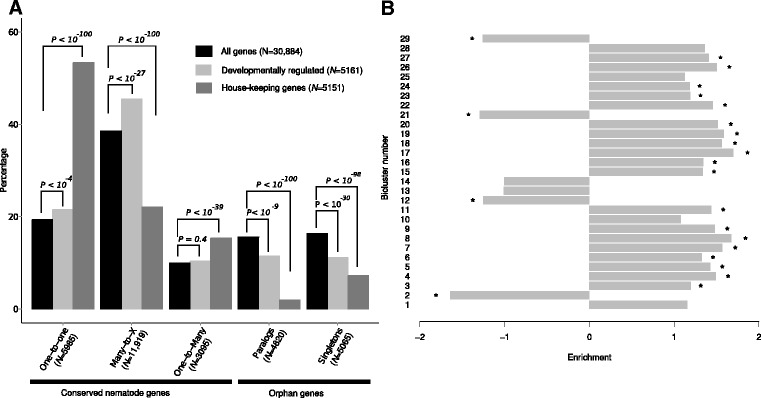
Enrichment of genes with paralogs among developmentally regulated genes. **a** Percentage of genes in each of the five classes, defined by orthology relationship and expression pattern. For developmentally regulated genes (union of all genes found in biclusters), the strongest enrichment consists in a significant overrepresentation (*P*<10^−27^) in conserved multicopy genes (many-to-X). **b** Enrichment in genes with paralogs among individual biclusters. Nineteen out of 29 biclusters show a significant enrichment in genes with paralogs

To test whether the pattern observed in the analysis of the HSP gene families represents a general trend in the evolution of developmental regulation in *P. pacificus*, we hypothesized that *P. pacificus* genes with paralogs should be enriched in the set of developmentally regulated biclusters. We therefore classified *P. pacificus* into genes with one-to-one orthologs in *C. elegans*, conserved genes (genes that have homologs in other nematodes), and orphan genes (genes without homolog in any other nematode outside the *Pristionchus* genus). We further subdivide conserved and orphan genes into single copy and multicopy genes based on the presence of intra-species paralogs (see *Methods*). Conserved multicopy genes (many-to-X) represent multiple paralogous *P. pacificus* genes that have homologs in other nematodes and have many-to-many, many-to-one, and many-to-zero orthology relationships [24] with respect to *C. elegans* genes. The five orthology classes were tested for enrichment and depletion among developmentally regulated genes (union of genes in all biclusters, all of which show differential expression across the developmental timecourse). In agreement with our hypothesis, we found that developmentally regulated genes are highly enriched (*P*<10^−27^) in conserved multicopy genes (Fig. 4a). When testing for enrichment of orthology classes in indivdual biclusters, we found that 19 out of 29 biclusters showed a significant enrichement in conserved multicopy genes (Fig. 4b). We interpret these findings as evidence that lineage-specific duplications of developmentally regulated genes have occurred repeatedly within the *Pristionchus* lineage. However, in contrast to the examples of HSP gene families, we did not observe a general trend towards depletion in one-to-one orthologs. Instead, we found a weaker but still significant enrichment of one-to-one orthologs in developmentally regulated genes (Fig. 4a), suggesting a certain degree of conservation of developmental gene expression. House-keeping genes are in contrast strongly enriched in one-to-one orthologs (*P*<10^−100^, Fig. 4a). This may reflect an evolutionary constraint acting against gene loss and gain, such as for members of protein complexes where duplications may affect the stoichiometry of complexes [35]. With respect to orphan genes, the most significant trend is a depletion of developmentally regulated genes among single copy orphan genes, which could suggest that these genes are rather constitutively expressed throughout larval development but also that overall low expression of orphan genes [36] limits the detection of significant differential expression for this gene set.

## Discussion

The *C. elegans* and *P. pacificus* model has been a fruitful system for comparative studies of developmental biology [8, 10]. However, very little was known about gene expression changes that guided development of *P. pacificus* larvae on a genome-wide scale. Despite the caveat, that our employed bleaching protocols only resulted in an imperfect synchronization of worm cultures (Fig. 1a–c), we were able to identify transcriptomes corresponding to stages J2 and J3 which represent the first expression profiling data for early larval stages of *P. pacificus*. Unsupervised biclustering of the expression data detected 29 biclusters which show developmental-stage specific regulation throughout the timecourse (Fig. 1d–f, Additional file 2: Figure S1). A complementary set of 5151 (17 %) potential house-keeping genes was identified that showed robust expression in all samples and that did not reveal any signal for significant differential expression in any pairwise comparison.

Previous comparative transcriptomic studies in *P. pacificus* and also in the parasitic nematode *Strongyloides stercoralis* have identified largely divergent gene expression patterns in dauer worms [9, 34]. These studies found that only a small set of dauer-specific genes is shared with *C. elegans*, and in some cases they also showed divergent expression profiles with respect to dauer-specific up and downregulation. One major drawback of such comparative transcriptomic studies is that they mostly focus on the expression of one-to-one orthologs between different species even though alternative approaches exist for comparisons involving paralogous groups [37]. One rational for using only one-to-one orthologs for comparisons, consists in the assumption that orthologous genes might perform more similar functions than genes that have undergone lineage-specific duplications. This assumption is commonly referred to as ortholog conjecture and its validity is still under debate [38]. However, the absence of one-to-one orthology might already reveal important evolutionary patterns. More precisely, lack of one-to-one orthology may be due to several reasons, such as gene duplication or loss in either of the lineages. To a certain extent other processes such as de novo gene formation, horizontal gene transfer, and rapid evolution may have contributed to missing homology and consequently also lack of orthology [39].

In this study, we characterized patterns of conservation within a broader developmental time-scale and in the case of heat shock proteins, we investigated the apparent divergence in expression profiles in greater detail. In both examined gene families (HSP20 and HSP70), we did not find any developmentally regulated gene with one-to-one ortholog in *C. elegans*. In contrast, we observed a tendency of developmentally regulated genes to occur in *P. pacificus*-specific subtrees suggesting that they had undergone duplication events since the separation from the *C. elegans* lineage. Even paralogous genes that were not captured as developmentally regulated due to missing significance in the differential expression analysis, showed very similar expression profiles as the developmentally regulated genes that were captured by our biclustering approach (Fig. 2b and c, Fig. 3b–d).

We examined these findings on a genome-wide scale by testing different orthology classes for enrichment in developmentally regulated genes (Fig. 4). Consistent with the case study of heat shock proteins, we found that the strongest enrichment in developmental regulation was observed for genes with putative paralogs, i.e. conserved multicopy genes (many-to-X category in Fig. 4). More precisely we found that 19 out of 29 biclusters showed a significant enrichment in conserved multicopy genes. This indicates that the developmental transcriptome of *P. pacificus* is shaped by ancient gene duplication events. Such ancient duplications may represent a plausible evolutionary mechanism to increase the dosage of developmentally regulated genes. The implicated positive selection on gene dosage has so far not explicitly been incorporated in previous models of gene duplication which assumed an initially neutral effect of duplication events [38, 40, 41] and focused on mechanisms that may give rise to neofunctionalization and subfunctionalization within a gene family. Such trends may be supported by the finding of dauer-specific and early larval-specific paralogous clusters in one gene family (Fig. 3b,c), as well as divergent expression profiles of genes within one paralogous cluster (Fig. 3d). However, further experimental and computational work is needed to allow a more detailed characterization of patterns of subfunctionalization in *P. pacificus*.

Similar findings have been obtained from a comparison of developmental transcriptomes of *C. elegans* and *C. briggsae* and other *Caenorhabditis* species, which revealed higher levels of conservation of genes expressed in early embryos when compared to later developmental stages and it was argued that this reflected a developmental constraint, i.e. selection against gene duplicates of genes expressed during embryogenesis [12, 14]. However, this explanation is not mutually exclusive with a scenario of increasing gene dosage by gene duplication at later stages of development. Also outside the nematode phylum, different studies in insects and vertebrates have shown that genes that are expressed in larvae and adults tend to be younger as opposed to genes that are specifically expressed at early embryogenesis [13, 42]. This pattern is also compatible with two previously proposed models of evolution of gene expression, the funnel and the hourglass model, which both predict a higher level of expression divergence at later developmental stages [43]. This general pattern might help to explain the substantial divergence observed in comparisons of expression profiles at larger time-scales [9, 34] and to improve our understanding of the evolution of developmental-stage specific expression in nematodes.

The comparison to *C. elegans* reveals trends that likely evolved in the range of hundreds of millions of years [7], but the *Pristionchus* system provides a powerful phylogenetic framework including roughly 30 known species [44] and hundreds of *P. pacificus* strains with available genomic data [45, 46] that allow to investigate the genetic architecture of transcriptional variation by expression quantitative trait loci and allele-specific expression studies [47]. Thus, future studies may further elucidate the genetic basis of changes in gene expression at much smaller evolutionary distance.

## Conclusion

By combining the investigation of developmental transcriptomes for the nematode *P. pacificus* with phylogenomic analyses, our study connects the evolution of gene expression, gene duplications, and development. The most striking pattern seems to be that most developmentally regulated genes are result of lineage-specific duplications. Such a trend could be explained by selection for higher gene dosage that drives the duplication of developmentally regulated genes. We speculate, this process represents an important factor in the genetic and phenotypic diversification of nematodes. Our findings may help to better interpret the relatively small proportion of highly conserved genes among developmentally regulated genes and to better understand the evolution of developmental-stage specific expression.

## Data availability

Raw reads have been submitted to the European Nucleotide Archive under the accession number PRJEB5534. Gene models (version TAU) are available at http://www.pristionchus.org/download/.

## Ethics statement

This study does not involve research on humans or human material and also not on animals according to the german animal protection legislation. Therefore no ethical approval is needed.

## Additional files

Additional file 1
**Table with expression fold changes and p-values.** Excel file with all expression FPKM values for all genes, fold changes and FDR corrected p-values for all genes that were found to be significantly differentially expressed in at least one comparison. The file also includes the assignments of genes to expression biclusters. (XLS 19558 kb)

Additional file 2
**Supplemental figures, tables, and data.** PDF file with supplemental figures, tables, and data. (PDF 800 kb)

## References

[1] Chalfie M, Sulston JE, White JG, Southgate E, Thomson JN, Brenner S (1985). The neural circuit for touch sensitivity in *Caenorhabditis elegans*. J Neurosci.

[2] Avery L, Horvitz HR (1989). Pharyngeal pumping continues after laser killing of the pharyngeal nervous system of *C. elegans*. Neuron.

[3] Hong RL, Sommer RJ (2006). *Pristionchus pacificus*: a well-rounded nematode. Bioessays.

[4] Ogawa A, Streit A, Antebi A, Sommer RJ (2009). A conserved endocrine mechanism controls the formation of dauer and infective larvae in nematodes. Curr Biol.

[5] Blaxter ML, De Ley P, Garey JR, Liu LX, Scheldeman P, Vierstraete A (1998). A molecular evolutionary framework for the phylum nematoda. Nature.

[6] Stein LD, Bao Z, Blasiar D, Blumenthal T, Brent MR, Chen N (2003). The genome sequence of *Caenorhabditis briggsae*: a platform for comparative genomics. PLoS Biol.

[7] Dieterich C, Clifton SW, Schuster LN, Chinwalla A, Delehaunty K, Dinkelacker I (2008). The *Pristionchus pacificus* genome provides a unique perspective on nematode lifestyle and parasitism. Nat Genet.

[8] Kienle S, Sommer RJ (2013). Cryptic variation in vulva development by cis-regulatory evolution of a hairy-binding site. Nat Commun.

[9] Sinha A, Sommer RJ, Dieterich C (2012). Divergent gene expression in the conserved dauer stage of the nematodes *Pristionchus pacificus* and *Caenorhabditis elegans*. BMC Genomics.

[10] Bumbarger DJ, Riebesell M, Rödelsperger C, Sommer RJ (2013). System-wide rewiring underlies behavioral differences in predatory and bacterial-feeding nematodes. Cell.

[11] Hill AA, Hunter CP, Tsung BT, Tucker-Kellogg G, Brown EL (2000). Genomic analysis of gene expression in *C. elegans*. Science.

[12] Levin M, Hashimshony T, Wagner F, Yanai I (2012). Developmental milestones punctuate gene expression in the *Caenorhabditis* embryo. Dev Cell.

[13] Domazet-Lošo T, Tautz D (2010). A phylogenetically based transcriptome age index mirrors ontogenetic divergence patterns. Nature.

[14] Castillo-Davis CI, Hartl DL (2002). Genome evolution and developmental constraint in *Caenorhabditis elegans*. Mol Biol Evol.

[15] Wolf YI, Carmel L, Koonin EV (2006). Unifying measures of gene function and evolution. Proc Biol Sci.

[16] Spencer WC, Zeller G, Watson JD, Henz SR, Watkins KL, McWhirter RD (2011). A spatial and temporal map of *C. elegans* gene expression. Genome Res.

[17] Trapnell C, Roberts A, Goff L, Pertea G, Kim D, Kelley DR (2012). Differential gene and transcript expression analysis of RNA-seq experiments with tophat and cufflinks. Nat Protoc.

[18] Tanay A, Sharan R, Shamir R (2002). Discovering statistically significant biclusters in gene expression data. Bioinformatics.

[19] Bose N, Meyer JM, Yim JJ, Mayer MG, Markov GV, Ogawa A (2014). Natural variation in dauer pheromone production and sensing supports intraspecific competition in nematodes. Curr Biol.

[20] Penkov S, Ogawa A, Schmidt U, Tate D, Zagoriy V, Boland S (2014). A wax ester promotes collective host finding in the nematode *Pristionchus pacificus*. Nat Chem Biol.

[21] Schuster LN, Sommer RJ (2012). Expressional and functional variation of horizontally acquired cellulases in the nematode *Pristionchus pacificus*. Gene.

[22] Mitreva M, Jasmer DP, Zarlenga DS, Wang Z, Abubucker S, Martin J (2011). The draft genome of the parasitic nematode *Trichinella spiralis*. Nat Genet.

[23] Remm M, Storm CE, Sonnhammer EL (2001). Automatic clustering of orthologs and in-paralogs from pairwise species comparisons. J Mol Biol.

[24] Jensen RA (2001). Orthologs and paralogs - we need to get it right. Genome Biol.

[25] Sievers F, Higgins DG (2014). Clustal omega, accurate alignment of very large numbers of sequences. Methods Mol Biol.

[26] Darriba D, Taboada GL, Doallo R, Posada D (2011). Prottest 3: fast selection of best-fit models of protein evolution. Bioinformatics.

[27] Schliep KP (2011). Phangorn: phylogenetic analysis in R. Bioinformatics.

[28] Mortazavi A, Williams BA, McCue K, Schaeffer L, Wold B (2008). Mapping and quantifying mammalian transcriptomes by RNA-seq. Nat Methods.

[29] Anavy L, Levin M, Khair S, Nakanishi N, Fernandez-Valverde SL, Degnan BM (2014). Blind ordering of large-scale transcriptomic developmental timecourses. Development.

[30] Huang DW, Sherman BT, Lempicki RA (2009). Bioinformatics enrichment tools: paths toward the comprehensive functional analysis of large gene lists. Nucleic Acids Res.

[31] Rae R, Sinha A, Sommer RJ (2012). Genome-wide analysis of germline signaling genes regulating longevity and innate immunity in the nematode *Pristionchus pacificus*. PLoS Pathog.

[32] Sinha A, Rae R, Iatsenko I, Sommer RJ (2012). System wide analysis of the evolution of innate immunity in the nematode model species *Caenorhabditis elegans* and *Pristionchus pacificus*. PLoS One.

[33] Bargmann CI. Chemosensation in *C. elegans*. WormBook. 2006:1–29. 10.1895/wormbook.1.123.1 PMC4781564 18050433

[34] Stoltzfus JD, Minot S, Berriman M, Nolan TJ, Lok JB (2012). RNA-seq analysis of the parasitic nematode *Strongyloides stercoralis* reveals divergent regulation of canonical dauer pathways. PLoS Negl Trop Dis.

[35] Gout JF, Kahn D, Duret L, Paramecium Post-Genomics Consortium (2010). The relationship among gene expression, the evolution of gene dosage, and the rate of protein evolution. PLoS Genet.

[36] Borchert N, Dieterich C, Krug K, Schz W, Jung S, Nordheim A (2010). Proteogenomics of *Pristionchus pacificus* reveals distinct proteome structure of nematode models. Genome Res.

[37] Kristiansson E, Österlund T, Gunnarsson L, Arne G, Larsson DGJ, Nerman O (2013). A novel method for cross-species gene expression analysis. BMC Bioinforma.

[38] Rogozin IB (2014). Complexity of gene expression evolution after duplication: protein dosage rebalancing. Genet Res Int.

[39] Rödelsperger C, Streit A, Sommer RJ. Structure, function and evolution of the nematode genome. eLS. 2013.

[40] Ohno S (1970). Evolution by Gene Duplication.

[41] Lynch M, Katju V (2004). The altered evolutionary trajectories of gene duplicates. Trends Genet.

[42] Yang L, Zou M, Fu B, He S (2013). Genome-wide identification, characterization, and expression analysis of lineage-specific genes within zebrafish. BMC Genomics.

[43] Irie N, Kuratani S (2011). Comparative transcriptome analysis reveals vertebrate phylotypic period during organogenesis. Nat Commun.

[44] Kanzaki N, Ragsdale EJ, Herrmann M, Sommer RJ (2014). Two new and two recharacterized species from a radiation of *Pristionchus* (nematoda: diplogastridae) in europe. J Nematol.

[45] Rödelsperger C, Neher RA, Weller AM, Eberhardt G, Witte H, Mayer WE (2014). Characterization of genetic diversity in the nematode *Pristionchus pacificus* from population-scale resequencing data. Genetics.

[46] Baskaran P, Rödelsperger C (2015). Microevolution of duplications and deletions and their impact on gene expression in the nematode *Pristionchus pacificus*. PLoS One.

[47] Emerson JJ, Li WH (2010). The genetic basis of evolutionary change in gene expression levels. Philos Trans R Soc Lond B Biol Sci.

