# Supplementary material for: Ancient gene duplications have shaped developmental stage-specific expression in Pristionchus pacificus
Source: BMC Evol Biol. 2015 Sep 15;15:185. doi: 10.1186/s12862-015-0466-2 (PMC4570658; doi:10.1186/s12862-015-0466-2)
Supplement: Additional file 2 — Supplemental figures, tables, and data. PDF file with supplemental figures, tables, and data. (PDF 800 kb) [file 12862_2015_466_MOESM2_ESM.pdf]

Supplementary Figures and Tables

Ancient gene duplications have shaped developmental stage-specific expression in *Pristionchus pacificus*

Praveen Baskaran[1], Christian Rödelsperger[1], Neel Prabh[1], Vahan Serobyan[1], Gabriel Markov[1], Antje Hirsekorn[2] and Christoph Dieterich[3]

1) Max-Planck Institute for Developmental Biology
   Spemannstr. 35-39
   72076 Tübingen

2) Max-Delbrück Center for Molecular Medicine
   Robert-Rössle-Straße 10
   13125 Berlin-Buch

3) Max-Planck Institute for Biology of Ageing
   Joseph-Stelzmann-Str. 9b
   50866 Köln

Supplemental Figure Legends

Figure S1 - Expression levels of bicluster genes across all transcriptomes.
Biclusters were identified by the program Samba based on data for significant differential expression (FDR<0.01, CuffDiff) for all pairwise comparisons. Gene expression levels were quantified as FPKM values using the software Cufflinks and for each bicluster, the distribution of expression levels is shown as violin plots across all ten samples. The order of samples was determined by the software Blind and the color codes indicates the rough grouping of samples into i) dauer larva (black), ii) early larvae (gray), and iii) adults and late larvae (white). All biclusters show highly significant differential expression (FDR<0.01, Wilcoxon test) in at least one comparison across the three developmentalgroupings. This supports the interpretation that all identified biclusters represent genes that are developmentally regulated.

Figure S2 – Comparison between RNA-seq and qRT-PCR.
We chose six candidate genes for qRT-PCR validation of expression values as obtained by RNA-seq. The left column shows the RNA-seq expression values (FPKM) in all ten transcriptomes, the right column shows the relative expression values as determined by qRT-PCR (mean and standard error for four biological replicates). The colour code indicates our previous grouping of transcriptomes into developmental stages (see Figure 1). With exception of the *P. pacificus* gene predictions Contig53-snapTAU.140, all genes showed very consistent expression values between both methods.

Supplemental Table Legends

Table S1 – Gene Ontology (GO) term enrichment
Overrepresentation of GO terms was done by borrowing annotations from *C. elegans*. For this purpose, *C. elegans* one-to-one orthologs of bicluster and housekeeping genes were tested for enrichment using the David functional annotation webtool using the total set of one-to-one orthologs as background. As some biclusters had only a few genes with one-to-one orthologs in *C. elegans*, only gene sets with at least 90 one-to-one orthologs were tested. Only GO terms with a fold enrichment greater or equal than 2 and an FDR corrected p-value below 0.01 are shown.

Table S2 – Comparisons with previous *P. pacificus* transcriptome profiles
Comparisons with previous *P.pacificus* trancriptome profiles were done to test for enrichment of biclustered genes in various experimental studies. The data used in this comparisons were differentially expressed gene lists from studies on dauer vs dauer exit worms, germline ablated worms and worms exposed to four different bacterial pathogens. P-values were computed using Fisher's exact test and FDR for multiple testing corrections. Only biclusters with enrichment value greater than 1 and p-value less than 0.01 are shown.

Table S3 – Protein domain enrichment in bicluster genes

P. pacificus gene predictions (version TAU) were annotated with PFAM domains using the HMMER 3.0 package. For each bicluster and also for housekeeping genes, overrepresentation of protein domains was tested using Fisher's exact test. Only enriched PFAM domains with FDR corrected p-value below 0.01 are shown.

Table S4 – Evaluation of orthology predictions for *C. elegans* genes with one-to-one orthologs

For 57 *C. elegans* genes, with one-to-one ortholog in *P. pacificus* (Supplementary Data 1), we tested whether the correct ortholog could identified using our automated one-to-one orthology prediction pipeline. The correct ortholog could indeed identified in 42 cases, failure in orthology assignment could in almost all cases be attributed to gene prediction errors (gene fusions, split predictions and missing predictions).

Table S5 - Evaluation of ortholog predictions for *C. elegans* genes without one-to-one orthologs

We used a data set of 50 genes, for which manual analysis could not identify *P. pacificus* orthologs, to test for false positive orthology predictions. In 48 out of 50 cases, no ortholog was predicted indicating a high reliability of our predictions.

Supplemental Data Legends

Supplementary Data S1 – Protein sequences for manually curated one-to-one orthologs

To assess the performance of our one-to-one orthology prediction pipeline, we compiled a set of 57 *C. elegans* genes with known *P. pacificus* one-to-one orthologs. The *P. pacificus* orthologs were identified by repeated rounds of multiple alignment with putative paralogs, maximum likelihood tree reconstruction, and manual curation (detection of split and fused gene predictions, completion using TBLASTN). These manually curated *P. pacificus* sequences were used for phylogenetic analysis only. Please note, that in most cases, they represent incomplete sequences as no experimental validation was carried out.

Figure S1

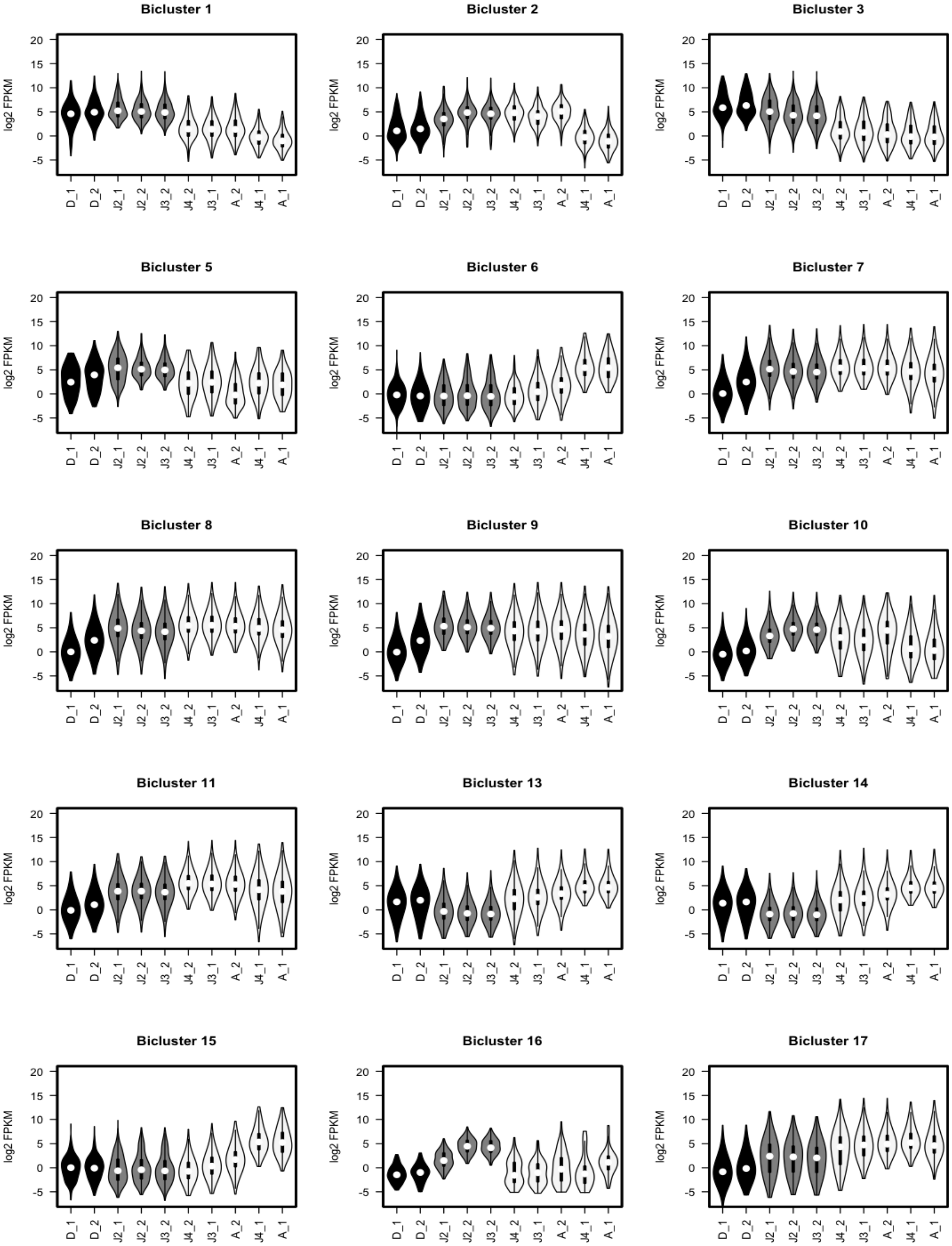
Bicluster 1
Bicluster 2
Bicluster 3
Bicluster 5
Bicluster 6
Bicluster 7
Bicluster 8
Bicluster 9
Bicluster 10
Bicluster 11
Bicluster 13
Bicluster 14
Bicluster 15
Bicluster 16
Bicluster 17
log2 FPKM
20
15
10
5
0
-5
D_1
D_2
J2_1
J2_2
J3_2
J4_2
J3_1
A_2
J4_1
A_1

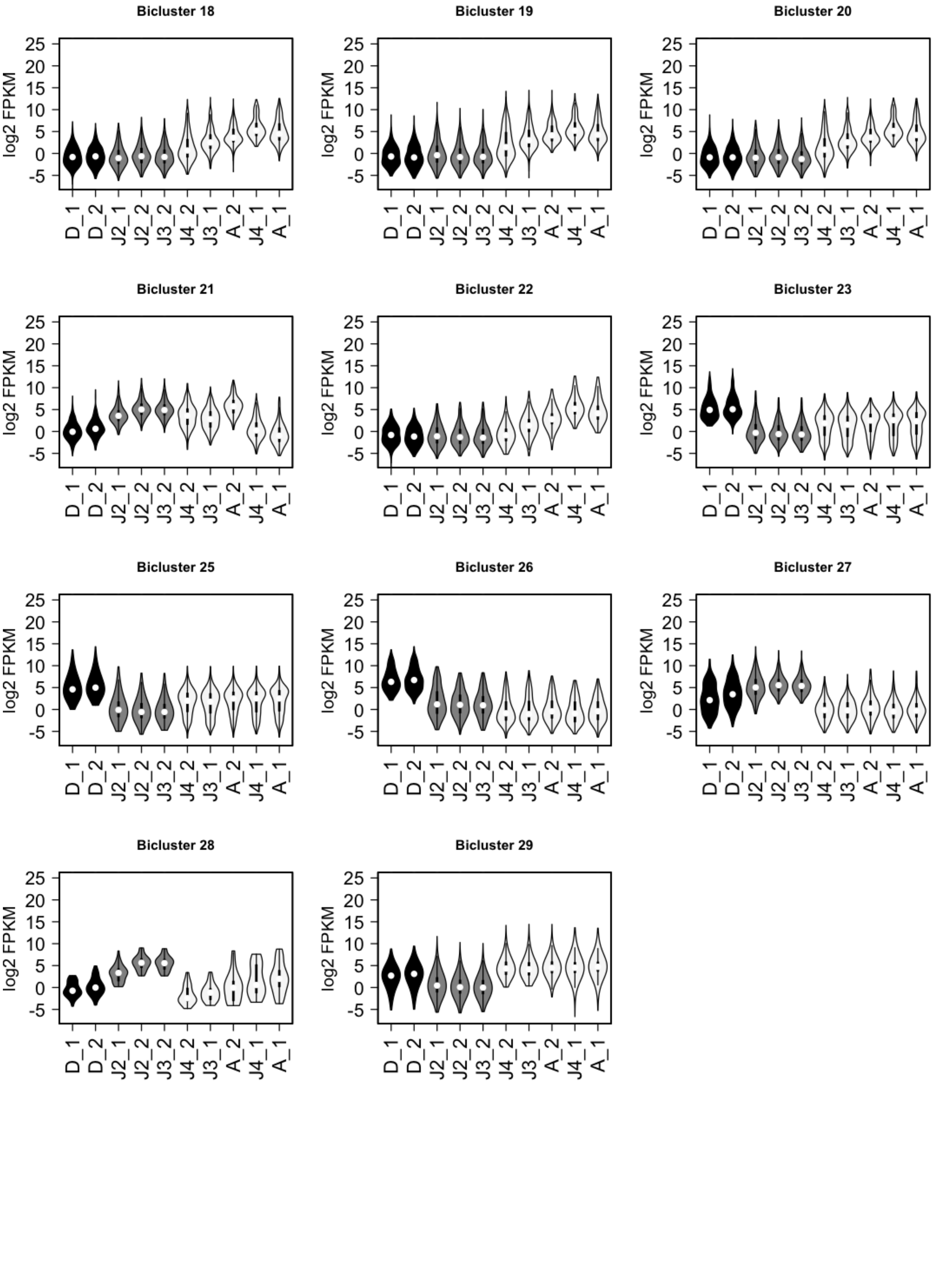
Bicluster 18
Bicluster 19
Bicluster 20
Bicluster 21
Bicluster 22
Bicluster 23
Bicluster 25
Bicluster 26
Bicluster 27
Bicluster 28
Bicluster 29
log2 FPKM
25
20
15
10
5
0
-5
D_1
D_2
J2_1
J2_2
J3_2
J4_2
J3_1
A_2
J4_1
A_1

Figure S2

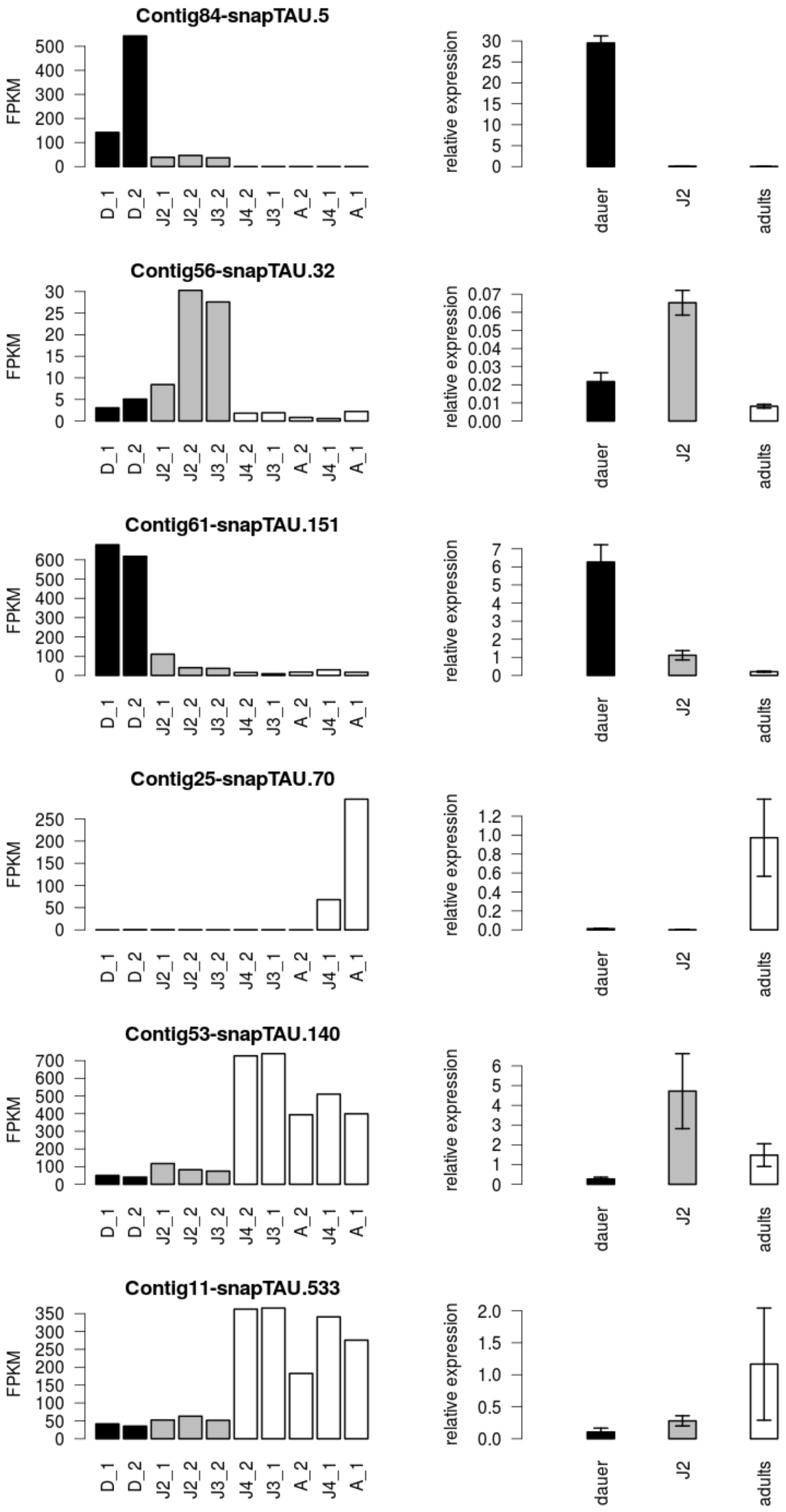
Contig84-snapTAU.5
Contig56-snapTAU.32
Contig61-snapTAU.151
Contig25-snapTAU.70
Contig53-snapTAU.140
Contig11-snapTAU.533
FPKM
relative expression
D_1
D_2
J2_1
J2_2
J3_2
J4_2
J3_1
A_2
J4_1
A_1
dauer
J2
adults

Table S1

Bicluster	GO_term	Description	Enrichment	FDR
1	GO:0030030	cell projection organization	10.7	0.008
2	GO:0042303	molting cycle	6.1	1.20E-08
2	GO:0018996	molting cycle- collagen and cuticulin-based cuticle	6.1	1.20E-08
2	GO:0018988	molting cycle- protein-based cuticle	6.1	1.20E-08
2	GO:0042302	structural constituent of cuticle	23.7	1.18E-05
2	GO:0008158	hedgehog receptor activity	16.2	2.8E-04
3	GO:0007218	neuropeptide signaling pathway	41.4	1.15E-14
3	GO:0007186	G-protein coupled receptor protein signaling pathway	10.1	5.61E-12
3	GO:0007166	cell surface receptor linked signal transduction	7.6	1.20E-09
7	GO:0044421	extracellular region part	15	4.85E-06
7	GO:0005578	proteinaceous extracellular matrix	15	4.18E-05
7	GO:0031012	extracellular matrix	14.4	5.75E-05
7	GO:0044420	extracellular matrix part	26.9	8.2E-04
7	GO:0005604	basement membrane	26.9	8.2E-04
7	GO:0005576	extracellular region	5.3	0.002
8	GO:0005578	proteinaceous extracellular matrix	21.4	1.80E-06
8	GO:0031012	extracellular matrix	20.7	2.50E-06
8	GO:0044421	extracellular region part	19.3	4.61E-06
8	GO:0005576	extracellular region	6.4	0.002
8	GO:0005604	basement membrane	32.2	0.007
8	GO:0044420	extracellular matrix part	32.2	0.007
10	GO:0018988	molting cycle- protein-based cuticle	6.2	8.84E-09
10	GO:0018996	molting cycle- collagen and cuticulin-based cuticle	6.2	8.84E-09
10	GO:0042303	molting cycle	6.2	8.84E-09
10	GO:0008158	hedgehog receptor activity	16.9	2.0E-04
10	GO:0005576	extracellular region	4.9	2.5E-04
21	GO:0018988	molting cycle- protein-based cuticle	6.5	1.01E-08
21	GO:0018996	molting cycle- collagen and cuticulin-based cuticle	6.5	1.01E-08
21	GO:0042303	molting cycle	6.5	1.01E-08
21	GO:0008158	hedgehog receptor activity	19.4	7.33E-05
21	GO:0006030	chitin metabolic process	26	0.002
21	GO:0006022	aminoglycan metabolic process	24	0.003
21	GO:0005576	extracellular region	4.6	0.003
24	GO:0007186	G-protein coupled receptor protein signaling pathway	9.5	9.07E-16
24	GO:0007218	neuropeptide signaling pathway	29.8	9.99E-13
24	GO:0007166	cell surface receptor linked signal transduction	7.1	1.29E-12
Housekeeping	GO:0030529	ribonucleoprotein complex	2.1	9.85E-32
Housekeeping	GO:0005840	ribosome	2.1	4.07E-24
Housekeeping	GO:0033279	ribosomal subunit	2.3	0.006

Table S2

Bicluster	Dauer Vs Dauer_exit				Germline ablation				Response to *Xenorhabdus nematophila*			
	Up (n= 3545)		Down (n= 1394)		Up (n= 994)		Down (n=2391)		Up (n= 848)		Down (n=4921)	
	Enrichment	pvalue	Enrichment	pvalue	Enrichment	pvalue	Enrichment	pvalue	Enrichment	pvalue	Enrichment	pvalue
1	2.9	4.4E-28	-	-	2.6	3.8E-06	-	-	6.1	1.7E-28	-	-
3	5.1	1.6E-93	-	-	3.5	3.3E-10	-	-	5.3	3.5E-20	-	-
4	2.5	2.3E-14	-	-	4.5	2.6E-14	-	-	7.7	1.1E -33	-	-
5	-	-	2.7	0.002	3.3	9.8E-04	-	-	8.7	2.7E-16	-	-
6	1.5	1.2E-04	-	-	5.1	1.3E-36	2.1	5.9E-11	-	-	2.3	2.2E-28
7	-	-	9.6	3.4E-272	2	8.5E-06	-	-	-	-	3.7	1.3E-146
8	-	-	10	3.7E-266	2.3	7.7E-09	-	-	-	-	4	2.0E-170
9	-	-	7.9	4.9E-197	2	1.6E-06	-	-	2.3	8.3E-09	2.8	2.6E-75
10	-	-	2.4	3.0E-12	-	-	-	-	-	-	1.3	0.005
11	-	-	8.5	2.5E-188	1.9	4.9E-05	-	-	-	-	3.5	1.8E-118
12	-	-	2.6	1.5E-17	1.9	5.3E-05	1.4	0.001	-	-	1.8	7.6E-17
13	-	-	1.4	8.55382E-04	4.3	2.7E-59	1.5	1.1E-07	1.9	2.4E-06	1.7	7.6E-20
14	-	-	-	-	4.5	4.5E-58	1.6	3.4E-09	1.9	2.3E-06	1.7	2.2E-17
15	-	-	-	-	5.4	2.8E-41	1.8	1.3E-07	-	-	2.1	4.3E-20
17	-	-	6.2	2.9E-86	7.3	2.9E-77	-	-	3.3	1.6E-14	3.1	5.7E-68
18	-	-	-	-	11.7	1.9E-102	-	-	5.3	3.2E-23	1.7	1.3E-06
19	-	-	-	-	10.6	4.4E-90	-	-	5.6	1.4E-26	1.6	1.2E-05
20	-	-	-	-	10.9	5.3E-102	-	-	5.4	1.3E-26	1.5	3.3E-05
22	-	-	-	-	13.3	6.9E-181	-	-	4.7	1.1E-25	-	-
23	3.9	4.8E-72	-	-	-	-	-	-	-	-	-	-
24	4.9	4.8E-124	-	-	2.9	3.5E-09	-	-	4.1	5.5E-18	-	-
25	3.7	1.7E-79	-	-	-	-	-	-	-	-	-	-
26	5.5	2.6E-69	-	-	2.6	5.2E-04	-	-	-	-	-	-
27	2.4	4.7E-13	-	-	4	9.19E-12	-	-	7.4	8.49E-33	-	-
29	-	-	3	3.1E-23	-	-	1.6	3.3E-05	-	-	2	7.2E-23

Biclu ster	Response *to Serratia marcescens*				Response to *Staphylococcus aureus*				Response to *Bacillus thuringiensis*			
	Up (n= 192)		Down (n=1006)		Up (n= 178)		Down (n= 140)		Up (n= 156)		Down (n=61)	
	Enrich ment	pvalue	Enrich ment	pvalue	Enrich ment	pvalue	Enrich ment	pvalue	Enrich ment	pvalue	Enrich ment	pvalue
1	5.5	2.1E-06	-	-	15	2.1E-26	-	-	6.3	1.7E-06	-	-
3	6.1	8.5E-07	-	-	13.1	7.1E-20	-	-	5	2.3E-04	-	-
4	9.5	1.6E-11	-	-	19.9	3.8E-31	-	-	11.7	6.6E-13	-	-
5	6.4	0.003	-	-	12.1	1.9E-06	-	-	7.9	0.002	15.2	0.001
6	-	-	4.3	4.7E-27	-	-	6.8	6.8E-10	-	-	-	-
7	5.2	2.8E-12	3.5	2.1E-26	5	1.1E-10	6.6	7.1E-14	7.6	3.0E-19	8.5	7.05E-10
8	5.2	1.8E-11	4.4	1.4E-39	5.4	2.1E-11	10.6	1.9E-27	6.4	1.5E-13	9.9	1.94E-11
9	5.2	8.7E-13	2.1	4.3E-08	5.6	1.3E-13	3.6	3.9E-05	9	4.0E-26	6.5	8.60E-07
10	-	-	-	-	-	-	4.4	9.1E-06	-	-	5.4	2.9E-04
11	3.7	3.8E-06	4.1	3.2E-32	2.6	0.003	12	6.9E-32	4.9	3.7E-08	18	2.1 E-26
12	-	-	3.9	1.2E-30	-	-	5.1	1.8E-08	-	-	-	-
13	-	-	3.5	2.4E-40	-	-	4.9	2.5E-12	-	-	-	-
14	-	-	3.4	2.8E-33	-	-	4.9	4.1E-11	-	-	-	-
15	-	-	3.8	1.7E-21	-	-	5.8	5.6E-08	-	-	-	-
17	2.9	0.0016 27387	4.4	2.6E-31	-	-	13.7	2.4E-32	3.2	0.001	14.1	2.1 E-15
18	-	-	2.7	4.7E-07	-	-	6.6	9.4E-07	-	-	-	-
19	-	-	2.6	1.0E-06	-	-	7	1.7E-07	-	-	-	-
20	-	-	2.5	1.6E-06	-	-	5.4	1.7E-05	-	-	-	-
21	-	-	-	-	-	-	4.5	0.001	-	-	-	-
24	3.9	1.5E-04	-	-	7.6	2.8E-12	-	-	-	-	-	-
27	7.5	2.6E-08	-	-	18.6	3.1E-29	-	-	10.6	1.52E-11	-	-
29	-	-	3.8	1.3E-27	-	-	5.4	6.2E-09	-	-	-	-

Table S3

Bicluster	PFAM domain	Domain Name	Count	Enrichment	FDR
1	PF00011.15	HSP20	9	31.9	1.35E-10
1	PF00012.14	HSP70	6	22.2	5.59E-06
1	PF06723.7	MreB_Mbl	5	23.4	3.97E-05
1	PF00211.14	Guanylate_cyc	6	11.1	3.15699E-04
1	PF00046.23	Homeobox	6	8.1	0.001774606
1	PF07700.9	HNOB	3	26.6	0.002567967
1	PF02782.10	FGGY_C	2	59.2	0.00510376
1	PF03226.8	Yippee	2	59.2	0.00510376
1	PF02932.10	Neur_chan_memb	6	5.7	0.008248486
2	PF00024.20	PAN_1	10	32.5	5.10E-12
2	PF02460.12	Patched	11	22.1	3.04E-11
2	PF00100.17	Zona_pellucida	10	27.2	3.04E-11
2	PF01079.14	Hint	7	54.2	1.81E-10
2	PF12349.2	Sterol-sensing	7	22.7	2.18E-07
2	PF00028.11	Cadherin	6	30.2	3.31E-07
2	PF01391.12	Collagen	12	6.4	4.37E-06
2	PF07062.6	Clc-like	3	50.3	1.72516E-04
2	PF12947.1	EGF_3	4	23.7	1.74891E-04
2	PF01683.12	EB	4	19.2	4.01141E-04
2	PF12799.1	LRR_4	5	11.2	6.26081E-04
2	PF00014.17	Kunitz_BPTI	4	12.6	0.001978596
2	PF01579.12	DUF19	2	67.1	0.001996034
2	PF01484.11	Col_cuticle_N	7	5.1	0.002965673
2	PF01697.21	DUF23	3	18.9	0.003059045
2	PF03176.9	MMPL	2	40.2	0.005471855
2	PF05762.8	VWA_CoxE	2	40.2	0.005471855
2	PF00008.21	EGF	4	8.9	0.005629952
2	PF00254.22	FKBP_C	2	33.5	0.00752665
3	PF00011.15	HSP20	6	23.2	3.88E-06
3	PF00211.14	Guanylate_cyc	7	14.1	1.17E-05
3	PF06723.7	MreB_Mbl	4	20.4	6.63926E-04
3	PF00106.19	adh_short	8	6	9.56285E-04
3	PF01094.22	ANF_receptor	5	11.3	0.001127355
3	PF00012.14	HSP70	4	16.1	0.001310604
3	PF00001.15	7tm_1	9	4.8	0.001426893
3	PF02782.10	FGGY_C	2	64.5	0.003531022
4	PF00012.14	HSP70	8	38.1	1.38E-09
4	PF06723.7	MreB_Mbl	7	42.1	7.65E-09
4	PF00011.15	HSP20	7	32	5.23E-08
4	PF03931.9	Skp1_POZ	4	14.8	0.004119506
4	PF01466.13	Skp1	4	13.9	0.004403621
4	PF02782.10	FGGY_C	2	76.3	0.004477601
4	PF00100.17	Zona_pellucida	4	12.4	0.00518635
4	PF01549.18	ShK	6	5.8	0.009421398

6	PF00635.20	Motile_Sperm	46	22.9	3.07E-49
6	PF00094.19	VWD	9	41.7	6.07E-13
6	PF09172.5	DUF1943	8	44.5	6.48E-12
6	PF00102.21	Y_phosphatase	11	7.9	1.72E-06
6	PF00149.22	Metallophos	11	6.7	1.00E-05
6	PF00642.18	zf-CCCH	5	17.4	7.70E-05
6	PF12150.2	MFP2b	3	41.7	2.39957E-04
6	PF02984.13	Cyclin_C	3	33.4	5.54866E-04
6	PF01391.12	Collagen	12	3.6	0.001526195
6	PF03057.8	DUF236	3	18.5	0.003925921
6	PF04156.8	IncA	3	16.7	0.005242424
6	PF01484.11	Col_cuticle_N	9	3.7	0.00679029
6	PF05611.5	DUF780	2	37.1	0.006862615
6	PF09811.3	Yae1_N	2	37.1	0.006862615
6	PF07714.1	Pkinase_Tyr	19	2.3	0.00729595
7	PF00059.15	Lectin_C	47	12.1	1.27E-36
7	PF01391.12	Collagen	36	7.1	3.11E-19
7	PF00092.22	VWA	20	13	2.49E-16
7	PF01484.11	Col_cuticle_N	27	7.3	7.69E-15
7	PF05577.6	Peptidase_S28	12	21.1	4.53E-13
7	PF05649.7	Peptidase_M13_N	9	17.5	6.97E-09
7	PF00431.14	CUB	15	7.4	1.68E-08
7	PF00450.16	Peptidase_S10	8	17.4	6.46E-08
7	PF01549.18	ShK	16	5	1.46E-06
7	PF01431.1	Peptidase_M13	9	9.2	4.00E-06
7	PF01079.14	Hint	6	17.1	5.96E-06
7	PF00026.17	Asp	9	8.7	6.15E-06
7	PF00052.12	Laminin_B	4	29.6	2.57E-05
7	PF00089.20	Trypsin	9	7.4	2.64E-05
7	PF06879.5	DUF1261	6	13	3.43E-05
7	PF00053.18	Laminin_EGF	5	16.8	5.04E-05
7	PF01764.1	Lipase_3	7	9.6	5.04E-05
7	PF01183.14	Glyco_hydro_25	4	24.6	6.48E-05
7	PF02520.11	DUF148	7	8.3	1.23854E-04
7	PF00090.13	TSP_1	5	14.2	1.23854E-04
7	PF00328.1	His_Phos_2	8	6.2	3.14156E-04
7	PF08445.4	FR47	5	10.3	6.96382E-04
7	PF00992.14	Troponin	4	14.8	7.27200E-04
7	PF00024.20	PAN_1	6	7.2	0.001151291
7	PF02055.10	Glyco_hydro_30	3	15.8	0.004493438
7	PF00743.13	FMO-like	4	9.2	0.005032417
7	PF07679.10	I-set	9	3.7	0.005032417
7	PF07859.7	Abhydrolase_3	8	4	0.0052213
7	PF00561.1	Abhydrolase_1	8	4	0.005558552
7	PF01757.16	Acyl_transf_3	6	5.3	0.005558552
7	PF00657.1	Lipase_GDSL	4	8.7	0.005903851
7	PF12697.1	Abhydrolase_6	9	3.4	0.008138594

7	PF00135.22	COesterase	9	3.4	0.008138594
7	PF00005.21	ABC_tran	9	3.3	0.009858654
8	PF00059.15	Lectin_C	47	13.2	3.15E-38
8	PF01391.12	Collagen	38	8.1	2.15E-22
8	PF01484.11	Col_cuticle_N	28	8.2	1.08E-16
8	PF00092.22	VWA	19	13.4	1.06E-15
8	PF05577.6	Peptidase_S28	11	21	8.34E-12
8	PF00431.14	CUB	16	8.5	5.49E-10
8	PF00450.16	Peptidase_S10	9	21.2	9.06E-10
8	PF05649.7	Peptidase_M13_N	9	19	3.15E-09
8	PF01549.18	ShK	17	5.8	7.25E-08
8	PF01431.1	Peptidase_M13	9	10	2.03E-06
8	PF00026.17	Asp	9	9.5	3.23E-06
8	PF00089.20	Trypsin	9	8	1.45E-05
8	PF06879.5	DUF1261	6	14.1	2.28E-05
8	PF01183.14	Glyco_hydro_25	4	26.7	5.28E-05
8	PF01764.1	Lipase_3	6	8.9	4.10996E-04
8	PF00657.1	Lipase_GDSL	5	11.8	4.10996E-04
8	PF00992.14	Troponin	4	16	6.19930E-04
8	PF00053.18	Laminin_EGF	4	14.6	9.26829E-04
8	PF00052.12	Laminin_B	3	24	0.001215475
8	PF00328.1	His_Phos_2	7	5.8	0.001406747
8	PF02798.14	GST_N	8	4.9	0.001611587
8	PF00090.13	TSP_1	4	12.3	0.001726488
8	PF07859.7	Abhydrolase_3	8	4.4	0.003419568
8	PF00561.1	Abhydrolase_1	8	4.3	0.003568423
8	PF02055.10	Glyco_hydro_30	3	17.2	0.003568423
8	PF01425.15	Amidase	4	10	0.00357545
8	PF00743.13	FMO-like	4	10	0.00357545
8	PF04101.10	Glyco_tran_28_C	10	3.5	0.00357545
8	PF00043.19	GST_C	7	4.8	0.00411496
8	PF01400.18	Astacin	7	4.7	0.004460358
8	PF00135.22	COesterase	9	3.7	0.004744465
8	PF00083.18	Sugar_tr	8	3.9	0.00655859
8	PF00337.16	Gal-bind_lectin	4	7.6	0.009370072
8	PF01433.1	Peptidase_M1	4	7.6	0.009370072
9	PF00059.15	Lectin_C	44	11	4.50E-32
9	PF00092.22	VWA	18	11.3	2.67E-13
9	PF00431.14	CUB	18	8.6	4.67E-11
9	PF05577.6	Peptidase_S28	8	13.7	1.20E-06
9	PF00450.16	Peptidase_S10	7	14.8	3.86E-06
9	PF01549.18	ShK	16	4.9	3.86E-06
9	PF05649.7	Peptidase_M13_N	7	13.2	8.90E-06
9	PF01484.11	Col_cuticle_N	16	4.2	2.55E-05
9	PF00992.14	Troponin	5	18	6.11E-05
9	PF06879.5	DUF1261	6	12.7	6.42E-05
9	PF00657.1	Lipase_GDSL	6	12.7	6.42E-05

9	PF01391.12	Collagen	18	3.4	8.24E-05
9	PF00201.12	UDPGT	16	3.5	1.72616E-04
9	PF01431.1	Peptidase_M13	7	7	6.64965E-04
9	PF00026.17	Asp	7	6.6	8.42841E-04
9	PF08768.5	DUF1794	3	26.9	8.42841E-04
9	PF04101.10	Glyco_tran_28_C	12	3.8	8.42841E-04
9	PF02798.14	GST_N	9	5	8.42841E-04
9	PF01674.12	Lipase_2	6	8	8.42841E-04
9	PF01764.1	Lipase_3	6	8	8.42841E-04
9	PF01757.16	Acyl_transf_3	7	6	0.00141029
9	PF00024.20	PAN_1	6	7	0.00173529
9	PF00089.20	Trypsin	7	5.6	0.002064286
9	PF01079.14	Hint	4	11	0.003055618
9	PF01183.14	Glyco_hydro_25	3	18	0.003428696
9	PF05497.6	Destabilase	3	15.4	0.005703036
9	PF03098.9	An_peroxidase	5	6.4	0.007426531
9	PF00112.17	Peptidase_C1	5	6.4	0.007426531
9	PF00043.19	GST_C	7	4.3	0.009288575
9	PF08445.4	FR47	4	8	0.009855853
10	PF00024.20	PAN_1	12	18.3	2.34E-11
10	PF01391.12	Collagen	24	6	6.20E-11
10	PF00100.17	Zona_pellucida	12	15.4	2.13E-10
10	PF01484.11	Col_cuticle_N	19	6.6	2.31E-09
10	PF01079.14	Hint	7	25.5	5.97E-08
10	PF00595.18	PDZ	12	7.8	7.92E-07
10	PF02460.12	Patched	9	8.5	1.66E-05
10	PF03931.9	Skp1_POZ	7	10.7	4.82E-05
10	PF12349.2	Sterol-sensing	7	10.7	4.82E-05
10	PF01466.13	Skp1	7	10	7.07E-05
10	PF03314.8	DUF273	5	16.9	9.54E-05
10	PF04155.12	Ground-like	7	9.5	9.54E-05
10	PF01682.13	DB	5	9.1	0.002335789
10	PF05649.7	Peptidase_M13_N	4	10	0.006992865
10	PF01683.12	EB	4	9	0.009944955
11	PF01391.12	Collagen	35	7.8	5.72E-20
11	PF01484.11	Col_cuticle_N	26	8	4.41E-15
11	PF00059.15	Lectin_C	26	7.7	1.23E-14
11	PF00092.22	VWA	13	9.6	1.12E-08
11	PF04101.10	Glyco_tran_28_C	16	6	1.86E-07
11	PF00450.16	Peptidase_S10	7	17.3	9.83E-07
11	PF00201.12	UDPGT	18	4.7	9.83E-07
11	PF05649.7	Peptidase_M13_N	7	15.5	2.17E-06
11	PF00100.17	Zona_pellucida	9	10.2	2.17E-06
11	PF01079.14	Hint	6	19.4	3.33E-06
11	PF00328.1	His_Phos_2	9	7.9	2.04E-05
11	PF02460.12	Patched	9	7.6	2.80E-05
11	PF03351.11	DOMON	5	17.6	5.76E-05

11	PF05577.6	Peptidase_S28	6	12	7.57E-05
11	PF00089.20	Trypsin	8	7.5	1.00046E-04
11	PF00431.14	CUB	10	5.6	1.10510E-04
11	PF01431.1	Peptidase_M13	7	8.2	1.83078E-04
11	PF00026.17	Asp	7	7.8	2.56764E-04
11	PF06879.5	DUF1261	5	12.4	3.28746E-04
11	PF00024.20	PAN_1	6	8.2	6.82156E-04
11	PF00005.21	ABC_tran	10	4.2	0.001251637
11	PF01073.13	3Beta_HSD	5	8.8	0.001742712
11	PF07993.6	NAD_binding_4	5	8.8	0.001742712
11	PF01183.14	Glyco_hydro_25	3	21.1	0.001970285
11	PF07690.10	MFS_1	16	2.8	0.002009605
11	PF01151.12	ELO	6	6.5	0.002166744
11	PF07859.7	Abhydrolase_3	8	4.6	0.002411908
11	PF01757.16	Acyl_transf_3	6	6	0.003116054
11	PF01549.18	ShK	10	3.6	0.003568384
11	PF00135.22	COesterase	9	3.9	0.003656621
11	PF00487.18	FA_desaturase	4	9.4	0.004826557
11	PF12349.2	Sterol-sensing	5	6.8	0.004831434
11	PF00664.17	ABC_membrane	7	4.6	0.004878888
11	PF00083.18	Sugar_tr	8	4.1	0.004878888
11	PF01266.18	DAO	4	8.9	0.005488873
11	PF04145.9	Ctr	3	14	0.005763546
11	PF01501.14	Glyco_transf_8	3	14	0.005763546
11	PF01370.15	Epimerase	6	5.1	0.006433166
11	PF02463.13	SMC_N	6	4.8	0.008581733
11	PF01579.12	DUF19	2	28.1	0.008806548
11	PF01156.13	IU_nuc_hydro	2	28.1	0.008806548
11	PF03015.13	Sterile	3	11.5	0.00991472
12	PF01391.12	Collagen	23	4.8	2.70E-09
12	PF01484.11	Col_cuticle_N	17	4.9	3.88E-07
12	PF02803.12	Thiolase_C	4	31.6	1.03E-05
12	PF00108.1	Thiolase_N	4	22.6	6.85E-05
12	PF00004.23	AAA	7	7.3	2.08111E-04
12	PF00400.26	WD40	11	4.2	3.05550E-04
12	PF07748.7	Glyco_hydro_38C	3	29.7	3.14198E-04
12	PF01400.18	Astacin	8	5.3	6.35864E-04
12	PF07724.8	AAA_2	4	12.2	0.001166041
12	PF00481.15	PP2C	4	12.2	0.001166041
12	PF00013.23	KH_1	5	7.9	0.001700798
12	PF02170.16	PAZ	4	10.5	0.00209655
12	PF03200.10	Glyco_hydro_63	3	16.9	0.002446884
12	PF04389.1	Peptidase_M28	3	14.8	0.00380483
12	PF11971.2	CAMSAP_CH	3	11.9	0.007703388
12	PF00890.18	FAD_binding_2	3	11.9	0.007703388
12	PF09261.5	Alpha-mann_mid	2	26.4	0.008047713
12	PF08954.5	DUF1900	2	26.4	0.008047713

12	PF09068.5	efhand_1	2	26.4	0.008047713
12	PF10513.3	EPL1	2	26.4	0.008047713
12	PF01571.15	GCV_T	2	26.4	0.008047713
12	PF06046.7	Sec6	2	26.4	0.008047713
12	PF02780.14	Transketolase_C	2	26.4	0.008047713
12	PF02171.11	Piwi	4	7.2	0.008742877
12	PF00173.22	Cyt-b5	3	10.8	0.009587389
13	PF00635.20	Motile_Sperm	30	6.6	5.03E-16
13	PF00102.21	Y_phosphatase	23	7.3	1.44E-13
13	PF07714.1	Pkinase_Tyr	52	2.7	3.11E-10
13	PF00094.19	VWD	9	18.4	3.19E-10
13	PF00069.19	Pkinase	54	2.6	1.45E-09
13	PF09172.5	DUF1943	8	19.6	1.64E-09
13	PF00149.22	Metallophos	20	5.3	3.68E-09
13	PF01391.12	Collagen	29	3.8	3.82E-09
13	PF01484.11	Col_cuticle_N	20	3.6	3.94E-06
13	PF00013.23	KH_1	8	7.8	2.20E-05
13	PF00017.18	SH2	10	5.5	5.34E-05
13	PF07748.7	Glyco_hydro_38C	3	18.4	0.001297009
13	PF12150.2	MFP2b	3	18.4	0.001297009
13	PF11971.2	CAMSAP_CH	4	9.8	0.002324349
13	PF02803.12	Thiolase_C	3	14.7	0.003064561
13	PF00004.23	AAA	7	4.5	0.003663905
13	PF00249.25	Myb_DNA-binding	3	12.3	0.005844706
13	PF00481.15	PP2C	4	7.5	0.006879868
13	PF03200.10	Glyco_hydro_63	3	10.5	0.009678228
13	PF00108.1	Thiolase_N	3	10.5	0.009678228
14	PF00102.21	Y_phosphatase	25	9	8.05E-17
14	PF00635.20	Motile_Sperm	28	6.9	1.96E-15
14	PF00094.19	VWD	9	20.7	1.15E-10
14	PF09172.5	DUF1943	8	22.1	6.70E-10
14	PF07714.1	Pkinase_Tyr	45	2.7	1.56E-08
14	PF00069.19	Pkinase	48	2.6	1.57E-08
14	PF00149.22	Metallophos	17	5.1	1.65E-07
14	PF01391.12	Collagen	22	3.2	7.56E-06
14	PF00013.23	KH_1	8	8.8	9.40E-06
14	PF01484.11	Col_cuticle_N	17	3.4	5.17E-05
14	PF07748.7	Glyco_hydro_38C	3	20.7	9.27438E-04
14	PF12150.2	MFP2b	3	20.7	9.27438E-04
14	PF00782.14	DSPc	6	5.3	0.003763403
14	PF00017.18	SH2	7	4.3	0.005406885
14	PF07423.5	DUF1510	3	11.8	0.007256981
14	PF02170.16	PAZ	4	7.4	0.008210232
15	PF00635.20	Motile_Sperm	53	25.6	2.78E-60
15	PF00094.19	VWD	9	40.5	7.66E-13
15	PF09172.5	DUF1943	8	43.2	8.00E-12
15	PF00149.22	Metallophos	12	7	1.76E-06

15	PF00642.18	zf-CCCH	5	16.9	9.42E-05
15	PF00102.21	Y_phosphatase	9	6.3	1.41287E-04
15	PF12150.2	MFP2b	3	40.5	2.60233E-04
15	PF01391.12	Collagen	13	3.7	5.22239E-04
15	PF03057.8	DUF236	3	18	0.004532047
15	PF00337.16	Gal-bind_lectin	4	10.3	0.004767409
15	PF04156.8	IncA	3	16.2	0.005746837
15	PF05611.5	DUF780	2	36	0.00770224
15	PF09811.3	Yae1_N	2	36	0.00770224
15	PF01484.11	Col_cuticle_N	9	3.5	0.007703324
16	PF00595.18	PDZ	9	29.1	1.25E-09
16	PF01466.13	Skp1	5	35.7	4.39E-06
16	PF03931.9	Skp1_POZ	5	38	4.39E-06
16	PF09757.3	Arb2	2	157.2	6.40993E-04
16	PF02172.10	KIX	2	78.6	0.002542623
17	PF01391.12	Collagen	32	8.6	2.71E-19
17	PF00149.22	Metallophos	18	9.9	5.89E-12
17	PF01484.11	Col_cuticle_N	21	7.7	8.56E-12
17	PF00059.15	Lectin_C	21	7.4	1.86E-11
17	PF00635.20	Motile_Sperm	18	8.1	1.52E-10
17	PF00092.22	VWA	10	8.9	2.65E-06
17	PF00102.21	Y_phosphatase	11	7.2	5.27E-06
17	PF00450.16	Peptidase_S10	6	17.8	8.72E-06
17	PF00069.19	Pkinase	28	2.7	2.45E-05
17	PF05577.6	Peptidase_S28	6	14.4	3.18E-05
17	PF07714.1	Pkinase_Tyr	25	2.7	9.42E-05
17	PF01151.12	ELO	7	9.1	1.18016E-04
17	PF00089.20	Trypsin	7	7.9	2.99263E-04
17	PF03015.13	Sterile	4	18.4	4.72638E-04
17	PF01073.13	3Beta_HSD	5	10.5	8.61057E-04
17	PF04101.10	Glyco_tran_28_C	10	4.5	8.61057E-04
17	PF07993.6	NAD_binding_4	5	10.5	8.61057E-04
17	PF05823.6	Gp-FAR-1	5	9.4	0.00149734
17	PF01400.18	Astacin	7	5.9	0.001533795
17	PF00201.12	UDPGT	11	3.4	0.003337059
17	PF05649.7	Peptidase_M13_N	4	10.6	0.003713069
17	PF07859.7	Abhydrolase_3	7	4.8	0.004519423
17	PF03057.8	DUF236	3	16.8	0.004519423
17	PF00026.17	Asp	5	6.7	0.006324055
17	PF00217.13	ATP-gua_Ptrans	2	33.7	0.007668524
17	PF02807.9	ATP-gua_PtransN	2	33.7	0.007668524
17	PF02009.10	Rifin_STEVOR	2	33.7	0.007668524
18	PF00635.20	Motile_Sperm	20	15.1	1.38E-16
18	PF00102.21	Y_phosphatase	16	17.6	2.00E-14
18	PF01391.12	Collagen	17	7.7	2.60E-09
18	PF00069.19	Pkinase	27	4.4	2.77E-09
18	PF07714.1	Pkinase_Tyr	25	4.5	7.07E-09

18	PF01484.11	Col_cuticle_N	14	8.6	1.39E-08
18	PF00149.22	Metallophos	12	11	1.39E-08
18	PF01347.16	Vitellogenin_N	4	48.4	8.02E-06
18	PF03057.8	DUF236	4	37.6	2.60E-05
18	PF12150.2	MFP2b	3	63.5	6.74E-05
18	PF00094.19	VWD	4	28.2	8.50E-05
18	PF09172.5	DUF1943	3	25.4	0.001662518
18	PF05823.6	Gp-FAR-1	4	12.5	0.002291812
18	PF00217.13	ATP-gua_Ptrans	2	56.4	0.002798656
18	PF02807.9	ATP-gua_PtransN	2	56.4	0.002798656
18	PF05677.6	DUF818	2	56.4	0.002798656
18	PF05884.6	ZYG-11_interact	2	56.4	0.002798656
18	PF00782.14	DSPc	4	10.9	0.003016188
18	PF02170.16	PAZ	3	16.9	0.00411328
18	PF01400.18	Astacin	5	7.1	0.004220624
19	PF01391.12	Collagen	31	13.5	1.89E-24
19	PF01484.11	Col_cuticle_N	23	13.7	2.47E-18
19	PF00635.20	Motile_Sperm	17	12.4	7.64E-13
19	PF00102.21	Y_phosphatase	14	14.9	9.34E-12
19	PF00069.19	Pkinase	30	4.8	3.46E-11
19	PF07714.1	Pkinase_Tyr	28	4.9	7.97E-11
19	PF00149.22	Metallophos	11	9.8	2.23E-07
19	PF05884.6	ZYG-11_interact	2	54.5	0.00516411
19	PF02170.16	PAZ	3	16.3	0.00800197
19	PF12150.2	MFP2b	2	40.9	0.00887854
20	PF00635.20	Motile_Sperm	20	13.6	1.20E-15
20	PF00069.19	Pkinase	37	5.5	2.04E-15
20	PF07714.1	Pkinase_Tyr	34	5.6	1.93E-14
20	PF00102.21	Y_phosphatase	16	15.8	8.42E-14
20	PF01391.12	Collagen	20	8.1	1.63E-11
20	PF01484.11	Col_cuticle_N	16	8.9	6.94E-10
20	PF00149.22	Metallophos	13	10.8	3.85E-09
20	PF00017.18	SH2	7	11.9	2.67E-05
20	PF12150.2	MFP2b	3	57.2	1.16011E-04
20	PF01347.16	Vitellogenin_N	3	32.7	9.09871E-04
20	PF03057.8	DUF236	3	25.4	0.001988423
20	PF12850.1	Metallophos_2	4	11.7	0.003627689
20	PF00094.19	VWD	3	19.1	0.004425216
20	PF05884.6	ZYG-11_interact	2	50.8	0.004711515
20	PF02170.16	PAZ	3	15.3	0.007900775
21	PF01391.12	Collagen	29	13.8	5.09E-23
21	PF01484.11	Col_cuticle_N	26	16.9	5.09E-23
21	PF00024.20	PAN_1	11	31.7	4.87E-13
21	PF00100.17	Zona_pellucida	11	26.5	3.86E-12
21	PF01079.14	Hint	7	48.1	5.69E-10
21	PF02460.12	Patched	9	16.1	6.18E-08
21	PF12349.2	Sterol-sensing	7	20.2	5.70E-07

21	PF04155.12	Ground-like	6	15.3	2.76E-05
21	PF01682.13	DB	5	17.2	9.97E-05
21	PF01683.12	EB	4	17	7.83685E-04
21	PF01273.19	LBP_BPI_CETP	3	24.3	0.001868004
21	PF01674.12	Lipase_2	4	13.2	0.001868004
21	PF01579.12	DUF19	2	59.5	0.002586779
21	PF00182.13	Glyco_hydro_19	2	59.5	0.002586779
21	PF06119.8	NIDO	2	59.5	0.002586779
21	PF00246.1	Peptidase_M14	3	19.1	0.002875213
21	PF00092.22	VWA	5	7.8	0.002875213
21	PF08768.5	DUF1794	2	44.6	0.003968089
21	PF02886.11	LBP_BPI_CETP_C	3	16.7	0.003968089
21	PF00629.17	MAM	2	44.6	0.003968089
21	PF12947.1	EGF_3	3	15.8	0.004327881
21	PF03176.9	MMPL	2	35.7	0.00601742
21	PF12799.1	LRR_4	4	7.9	0.007580133
22	PF00635.20	Motile_Sperm	40	21.6	4.33E-41
22	PF00149.22	Metallophos	23	15.1	1.44E-19
22	PF07714.1	Pkinase_Tyr	42	5.5	1.06E-17
22	PF00069.19	Pkinase	44	5.2	1.10E-17
22	PF00102.21	Y_phosphatase	17	13.3	1.18E-13
22	PF00017.18	SH2	11	14.8	1.89E-09
22	PF00094.19	VWD	6	30.2	2.08E-07
22	PF01347.16	Vitellogenin_N	5	43.2	2.80E-07
22	PF09172.5	DUF1943	5	30.2	2.97E-06
22	PF12150.2	MFP2b	3	45.3	1.71563E-04
22	PF12850.1	Metallophos_2	5	11.6	5.40089E-04
22	PF01391.12	Collagen	12	3.9	6.41207E-04
22	PF03057.8	DUF236	3	20.1	0.00278889
22	PF00217.13	ATP-gua_Ptrans	2	40.3	0.005211651
22	PF02807.9	ATP-gua_PtransN	2	40.3	0.005211651
22	PF09811.3	Yae1_N	2	40.3	0.005211651
22	PF05884.6	ZYG-11_interact	2	40.3	0.005211651
22	PF01734.1	Patatin	3	13.9	0.006984108
22	PF00595.18	PDZ	6	5	0.007803831
22	PF00782.14	DSPc	4	7.8	0.009198866
23	PF00067.16	p450	13	4.2	2.26306E-04
23	PF01323.14	DSBA	3	25.2	0.002209678
23	PF04116.7	FA_hydroxylase	3	25.2	0.002209678
23	PF00501.22	AMP-binding	6	6.8	0.002933569
23	PF01151.12	ELO	5	8.6	0.003116413
23	PF01756.13	ACOX	3	18.4	0.005438884
23	PF09068.5	efhand_1	2	44.9	0.006628811
23	PF06046.7	Sec6	2	44.9	0.006628811
24	PF00001.15	7tm_1	16	6	3.70E-07
24	PF00011.15	HSP20	7	18.9	1.31E-06
24	PF00067.16	p450	14	4.5	6.39E-05

24	PF04800.6	ETC_C1_NDUFA4	3	40.5	5.90610E-04
24	PF00211.14	Guanylate_cyc	6	8.4	0.001273642
24	PF00106.19	adh_short	9	4.7	0.002157427
24	PF01094.22	ANF_receptor	5	7.8	0.006217767
24	PF00027.23	cNMP_binding	3	16.9	0.008351941
24	PF02782.10	FGGY_C	2	45	0.008351941
25	PF00067.16	p450	16	4.2	2.23E-05
25	PF01151.12	ELO	6	8.3	8.63221E-04
25	PF01323.14	DSBA	3	20.3	0.003484518
25	PF04116.7	FA_hydroxylase	3	20.3	0.003484518
25	PF12697.1	Abhydrolase_6	8	4.4	0.004879348
25	PF00501.22	AMP-binding	6	5.5	0.007564589
25	PF01756.13	ACOX	3	14.8	0.008618832
25	PF00201.12	UDPGT	10	3.3	0.008618832
25	PF09068.5	efhand_1	2	36.1	0.008832611
25	PF06046.7	Sec6	2	36.1	0.008832611
25	PF00011.15	HSP20	4	8.7	0.009115183
26	PF00106.19	adh_short	7	8.3	7.68058E-04
26	PF01151.12	ELO	4	15.7	0.002992065
26	PF00001.15	7tm_1	7	5.9	0.003417349
26	PF00487.18	FA_desaturase	3	25.5	0.003417349
26	PF08659.4	KR	5	8.2	0.005198676
26	PF00067.16	p450	7	5.1	0.005640403
27	PF00012.14	HSP70	8	36.8	9.78E-10
27	PF06723.7	MreB_Mbl	7	40.6	6.26E-09
27	PF00011.15	HSP20	6	26.5	1.96E-06
27	PF03931.9	Skp1_POZ	4	14.2	0.003578321
27	PF02782.10	FGGY_C	2	73.5	0.003962111
27	PF01466.13	Skp1	4	13.4	0.003962111
27	PF00100.17	Zona_pellucida	4	11.9	0.005037857
28	PF00595.18	PDZ	4	40.3	3.30E-05
29	PF01391.12	Collagen	20	4.5	1.38E-07
29	PF01484.11	Col_cuticle_N	16	4.9	9.37E-07
29	PF02803.12	Thiolase_C	4	33.8	8.37E-06
29	PF00400.26	WD40	12	4.9	3.25E-05
29	PF00108.1	Thiolase_N	4	24.2	5.52E-05
29	PF00004.23	AAA	7	7.8	1.42327E-04
29	PF07748.7	Glyco_hydro_38C	3	31.7	2.72459E-04
29	PF07724.8	AAA_2	4	13	9.63396E-04
29	PF00481.15	PP2C	4	13	9.63396E-04
29	PF08371.5	PLD_envelope	3	21.2	0.001259916
29	PF02037.21	SAP	3	21.2	0.001259916
29	PF00076.16	RRM_1	11	3.6	0.001272641
29	PF01151.12	ELO	6	6.5	0.001418658
29	PF07728.8	AAA_5	5	8.1	0.001552852
29	PF03200.10	Glyco_hydro_63	3	18.1	0.002079418
29	PF11971.2	CAMSAP_CH	3	12.7	0.006628013

29	PF00890.18	FAD_binding_2	3	12.7	0.006628013
29	PF09261.5	Alpha-mann_mid	2	28.2	0.007538328
29	PF09068.5	efhand_1	2	28.2	0.007538328
29	PF10513.3	EPL1	2	28.2	0.007538328
29	PF02780.14	Transketolase_C	2	28.2	0.007538328
29	PF00173.22	Cyt-b5	3	11.5	0.008535534
HouseKeeping	PF00076.16	RRM_1	66	3.1	4.15E-19
HouseKeeping	PF00271.25	Helicase_C	45	3.5	2.70E-16
HouseKeeping	PF00226.25	DnaJ	24	5	2.00E-14
HouseKeeping	PF08477.7	Miro	45	3.1	7.23E-14
HouseKeeping	PF00071.16	Ras	48	2.8	1.15E-12
HouseKeeping	PF04670.6	Gtr1_RagA	29	3.8	4.55E-12
HouseKeeping	PF00025.15	Arf	46	2.7	2.25E-11
HouseKeeping	PF00179.1	UQ_con	19	4.6	1.99E-10
HouseKeeping	PF00400.26	WD40	45	2.6	2.10E-10
HouseKeeping	PF00022.13	Actin	13	5.6	1.75E-09
HouseKeeping	PF00009.21	GTP_EFTU	29	3.2	1.92E-09
HouseKeeping	PF07653.11	SH3_2	23	3.6	2.76E-09
HouseKeeping	PF01423.16	LSM	14	4.9	1.01E-08
HouseKeeping	PF00018.22	SH3_1	24	3.3	1.18E-08
HouseKeeping	PF00240.17	ubiquitin	18	4	1.89E-08
HouseKeeping	PF12847.1	Methyltransf_18	21	3.5	3.62E-08
HouseKeeping	PF08242.6	Methyltransf_12	13	4.9	4.97E-08
HouseKeeping	PF01926.17	MMR_HSR1	15	4.3	7.98E-08
HouseKeeping	PF11976.2	Rad60-SLD	15	4.3	7.98E-08
HouseKeeping	PF00153.21	Mito_carr	21	3.3	1.32E-07
HouseKeeping	PF00118.18	Cpn60_TCP1	12	4.8	2.42E-07
HouseKeeping	PF00808.17	CBFD_NFYB_HMF	18	3.4	8.04E-07
HouseKeeping	PF09439.4	SRPRB	15	3.6	2.44E-06
HouseKeeping	PF00578.15	AhpC-TSA	11	4.4	3.71E-06
HouseKeeping	PF08241.6	Methyltransf_11	16	3.3	5.02E-06
HouseKeeping	PF01585.17	G-patch	10	4.6	5.51E-06
HouseKeeping	PF00535.20	Glycos_transf_2	9	4.9	7.52E-06
HouseKeeping	PF00702.1	Hydrolase	17	3.1	8.55E-06
HouseKeeping	PF08282.6	Hydrolase_3	8	5.3	8.67E-06
HouseKeeping	PF07717.10	OB_NTP_bind	8	5.3	8.67E-06
HouseKeeping	PF08534.4	Redoxin	8	5.3	8.67E-06
HouseKeeping	PF00122.14	E1-E2_ATPase	15	3.3	9.19E-06
HouseKeeping	PF00782.14	DSPc	16	3.1	1.57E-05
HouseKeeping	PF00170.15	bZIP_1	13	3.5	1.59E-05
HouseKeeping	PF07716.9	bZIP_2	15	3.2	1.67E-05
HouseKeeping	PF00125.18	Histone	21	2.6	1.82E-05
HouseKeeping	PF01399.21	PCI	9	4.5	2.53E-05
HouseKeeping	PF03357.15	Snf7	9	4.5	2.53E-05
HouseKeeping	PF00270.23	DEAD	34	2.1	2.82E-05
HouseKeeping	PF01217.14	Clat_adaptor_s	8	4.8	3.65E-05
HouseKeeping	PF01679.11	Pmp3	8	4.8	3.65E-05

HouseKeeping	PF05175.8	MTS	10	4	4.11E-05
HouseKeeping	PF03946.8	Ribosomal_L11_N	11	3.7	5.17E-05
HouseKeeping	PF12710.1	HAD	14	3.1	5.37E-05
HouseKeeping	PF00505.13	HMG_box	14	3.1	5.37E-05
HouseKeeping	PF00160.15	Pro_isomerase	12	3.4	5.65E-05
HouseKeeping	PF02421.12	FeoB_N	9	4.2	6.93E-05
HouseKeeping	PF00298.13	Ribosomal_L11	9	4.2	6.93E-05
HouseKeeping	PF00004.23	AAA	17	2.7	8.89E-05
HouseKeeping	PF00171.1	Aldedh	10	3.7	9.25E-05
HouseKeeping	PF05739.13	SNARE	8	4.4	1.13138E-04
HouseKeeping	PF02969.11	TAF	8	4.4	1.13138E-04
HouseKeeping	PF08449.5	UAA	8	4.4	1.13138E-04
HouseKeeping	PF00036.26	efhand	20	2.4	1.38921E-04
HouseKeeping	PF01553.15	Acyltransferase	9	3.9	1.63866E-04
HouseKeeping	PF00892.14	EamA	9	3.9	1.63866E-04
HouseKeeping	PF03144.19	GTP_EFTU_D2	9	3.9	1.63866E-04
HouseKeeping	PF00397.20	WW	9	3.9	1.63866E-04
HouseKeeping	PF03114.12	BAR	7	4.7	1.73766E-04
HouseKeeping	PF01105.18	EMP24_GP25L	7	4.7	1.73766E-04
HouseKeeping	PF01060.17	DUF290	29	2	1.82009E-04
HouseKeeping	PF01656.17	CbiA	6	5.1	2.37339E-04
HouseKeeping	PF03637.11	Mob1_phocein	6	5.1	2.37339E-04
HouseKeeping	PF00753.21	Lactamase_B	8	4	2.86736E-04
HouseKeeping	PF00515.22	TPR_1	11	3.1	3.44616E-04
HouseKeeping	PF00085.14	Thioredoxin	24	2.1	3.97094E-04
HouseKeeping	PF00690.20	Cation_ATPase_N	7	4.2	4.91333E-04
HouseKeeping	PF04408.17	HA2	7	4.2	4.91333E-04
HouseKeeping	PF00992.14	Troponin	7	4.2	4.91333E-04
HouseKeeping	PF00439.19	Bromodomain	9	3.4	6.70598E-04
HouseKeeping	PF12763.1	efhand_3	9	3.4	6.70598E-04
HouseKeeping	PF01576.13	Myosin_tail_1	9	3.4	6.70598E-04
HouseKeeping	PF00642.18	zf-CCCH	9	3.4	6.70598E-04
HouseKeeping	PF01193.18	RNA_pol_L	6	4.5	8.05287E-04
HouseKeeping	PF00899.15	ThiF	6	4.5	8.05287E-04
HouseKeeping	PF03151.10	TPT	6	4.5	8.05287E-04
HouseKeeping	PF04851.9	ResIII	13	2.6	9.82929E-04
HouseKeeping	PF00628.23	PHD	14	2.5	0.001133673
HouseKeeping	PF10591.3	SPARC_Ca_bdg	7	3.8	0.00114498
HouseKeeping	PF02737.12	3HCDH_N	5	5	0.001201541
HouseKeeping	PF00006.19	ATP-synt_ab	5	5	0.001201541
HouseKeeping	PF00306.21	ATP-synt_ab_C	5	5	0.001201541
HouseKeeping	PF02353.14	CMAS	5	5	0.001201541
HouseKeeping	PF04969.10	CS	5	5	0.001201541
HouseKeeping	PF00180.14	Iso_dh	5	5	0.001201541
HouseKeeping	PF00549.13	Ligase_CoA	5	5	0.001201541
HouseKeeping	PF08700.5	Vps51	5	5	0.001201541
HouseKeeping	PF01775.11	Ribosomal_L18ae	8	3.4	0.001241402

HouseKeeping	PF00412.16	LIM	13	2.5	0.001421543
HouseKeeping	PF00378.14	ECH	11	2.7	0.001463773
HouseKeeping	PF00620.21	RhoGAP	11	2.7	0.001463773
HouseKeeping	PF00689.15	Cation_ATPase_C	8	3.2	0.002262917
HouseKeeping	PF05496.6	RuvB_N	8	3.2	0.002262917
HouseKeeping	PF12799.1	LRR_4	16	2.1	0.003575063
HouseKeeping	PF01067.16	Calpain_III	5	4.3	0.003576762
HouseKeeping	PF01417.14	ENTH	5	4.3	0.003576762
HouseKeeping	PF00626.16	Gelsolin	5	4.3	0.003576762
HouseKeeping	PF10559.3	Plug_translocon	5	4.3	0.003576762
HouseKeeping	PF00652.16	Ricin_B_lectin	5	4.3	0.003576762
HouseKeeping	PF01163.16	RIO1	5	4.3	0.003576762
HouseKeeping	PF02036.11	SCP2	5	4.3	0.003576762
HouseKeeping	PF01204.12	Trehalase	5	4.3	0.003576762
HouseKeeping	PF00152.14	tRNA-synt_2	5	4.3	0.003576762
HouseKeeping	PF00248.15	Aldo_ket_red	8	3	0.003831073
HouseKeeping	PF00176.17	SNF2_N	10	2.6	0.004072627
HouseKeeping	PF00928.15	Adap_comp_sub	6	3.6	0.004335035
HouseKeeping	PF00038.15	Filament	6	3.6	0.004335035
HouseKeeping	PF00587.19	tRNA-synt_2b	6	3.6	0.004335035
HouseKeeping	PF00307.25	CH	11	2.4	0.00463484
HouseKeeping	PF00155.15	Aminotran_1_2	9	2.7	0.005043339
HouseKeeping	PF01556.12	DnaJ_C	4	4.8	0.005872396
HouseKeeping	PF01207.11	Dus	4	4.8	0.005872396
HouseKeeping	PF00676.14	E1_dh	4	4.8	0.005872396
HouseKeeping	PF02781.1	G6PD_C	4	4.8	0.005872396
HouseKeeping	PF03143.11	GTP_EFTU_D3	4	4.8	0.005872396
HouseKeeping	PF00183.12	HSP90	4	4.8	0.005872396
HouseKeeping	PF03810.13	IBN_N	4	4.8	0.005872396
HouseKeeping	PF00459.19	Inositol_P	4	4.8	0.005872396
HouseKeeping	PF04193.8	PQ-loop	4	4.8	0.005872396
HouseKeeping	PF00428.13	Ribosomal_60s	4	4.8	0.005872396
HouseKeeping	PF00573.16	Ribosomal_L4	4	4.8	0.005872396
HouseKeeping	PF12718.1	Tropomyosin_1	4	4.8	0.005872396
HouseKeeping	PF08389.6	Xpo1	4	4.8	0.005872396
HouseKeeping	PF00293.1	NUDIX	7	3	0.007211516
HouseKeeping	PF02874.17	ATP-synt_ab_N	5	3.7	0.008071201
HouseKeeping	PF00648.1	Peptidase_C2	6	3.3	0.008071201

Table S4

C. elegans gene	one-to-one predictions	Correct
egl-9	Contig0-snapTAU.355	Yes
mod-5	Contig100-snapTAU.64	Yes
unc-129	Contig104-snapTAU.58	Yes
tax-4	Contig11-snapTAU.113	Yes
unc-58	Contig124-snapTAU.35	Yes
sma-2	Contig13-snapTAU.276	Yes
C34E11.2	Contig136-snapTAU.22	Yes
hst-3.2	Contig136-snapTAU.4	Yes
glb-5	Contig14-snapTAU.341	Yes
hsf-1	Contig147-snapTAU.20	Yes
ser-3	Contig16-snapTAU.25	Yes
hst-1	Contig177-snapTAU.2	Yes
mrp-4	Contig18-snapTAU.104	Yes
hsp-3	Contig19-snapTAU.321	Yes
sulp-1	Contig2-snapTAU.570	Yes
sulp-8	Contig20-snapTAU.200	Yes
tax-2	Contig21-snapTAU.118	Yes
ser-5	Contig22-snapTAU.314	Yes
cey-1	Contig24-snapTAU.1	Yes
exp-2	Contig254-snapTAU.2	Yes
zig-1	Contig3-snapTAU.69	Yes
mod-1	Contig30-snapTAU.105	Yes
sulp-7	Contig32-snapTAU.198	Yes
ser-7	Contig32-snapTAU.57	Yes
unc-119	Contig35-snapTAU.320	Yes
glr-1	Contig35-snapTAU.68	Yes
nfi-1	Contig36-snapTAU.133	Yes
mrp-5	Contig37-snapTAU.41	Yes
ser-1	Contig44-snapTAU.191	Yes
amx-1	Contig44-snapTAU.6	Yes
sma-4	Contig46-snapTAU.110	Yes
kel-8	Contig47-snapTAU.100	Yes
hif-1	Contig47-snapTAU.13	Yes
hst-3.1	Contig5-snapTAU.500	Yes
glr-7	Contig50-snapTAU.146	Yes
hst-2	Contig56-snapTAU.118	Yes
pah-1	Contig6-snapTAU.98	Yes
sul-3	Contig63-snapTAU.14	Yes
T14G8.3	Contig63-snapTAU.63	Yes
cat-2	Contig7-snapTAU.395	Yes
ser-2	Contig8-snapTAU.269	Yes
F37D6.6	Contig95-snapTAU.35	Yes

glt-1	not found	No
ser-4	not found	No
ser-6	not found	No
ocr-3	not found	No
dbl-1	not found	No
sma-3	not found	No
daf-8	not found	No
npr-1	not found	No
C17G1.7	not found	No
gcy-35	not found	No
gcy-36	not found	No
che-1	not found	No
mrp-3	not found	No
kvs-1	not found	No
tph-1	not found	No

Table S5

C. elegans gene	one-to-one predictions	Correct
ocr-2	Contig0-snapTAU.716	No
kel-3	Contig2-snapTAU.318	No
pxn-1	Contig42-snapTAU.152	No
kel-1	not found	Yes
phy-4	not found	Yes
cey-2	not found	Yes
cey-3	not found	Yes
fat-2	not found	Yes
sulp-3	not found	Yes
sulp-4	not found	Yes
sulp-5	not found	Yes
gcy-34	not found	Yes
gcy-32	not found	Yes
cep-1	not found	Yes
hsp-16.11	not found	Yes
hsp-16.48	not found	Yes
hsp-16.1	not found	Yes
hsp-16.49	not found	Yes
hsp-16.41	not found	Yes
hsp-16.2	not found	Yes
hpo-15	not found	Yes
lsd-1	not found	Yes
spr-5	not found	Yes
F55C5.6	not found	Yes
amx-3	not found	Yes

R08F11.7	not found	Yes
F09F3.5	not found	Yes
duox-2	not found	Yes
bli-3	not found	Yes
pxn-2	not found	Yes
K10B4.1	not found	Yes
C18B2.1	not found	Yes
C18B2.2	not found	Yes
F59D12.3	not found	Yes
C41C4.1	not found	Yes
F36D1.8	not found	Yes
F56H6.13	not found	Yes
F40C5.1	not found	Yes
T24A6.16	not found	Yes
F17B5.4	not found	Yes
ZK1025.2	not found	Yes
F49D11.3	not found	Yes
F49D11.6	not found	Yes
T15D6.1	not found	Yes
T09E11.3	not found	Yes
T27C5.12	not found	Yes
K06H6.5	not found	Yes
C31B8.9	not found	Yes
K07H8.8	not found	Yes
sul-2	not found	Yes

Data S1

```
>ppa_mod-5
KCFSQFEFLLAVVGYAVDLGNIWRFPTVCYKHGGGAFLIPWIVMLFLGGLPMFYMELALG
QFHRSGCISIWKRICPAFKGIGYGICFICTFIACFYNAIIARAVYFAFASFASLIFEWEV
PWKTCNNTWNTENCTETLDAMLMGNSSAWRKSPSEEYFQMHKSNGFDQLGGIKPSLAFCL
FIVFVMVYFALWKGPKSSGKVWSAAASQIFFSLGPGFGVLLALSSYNDFNNNCYRDALVT
SFINCATSFFSGFVIFSTLGYMSVLTNKPVDKVVEGADDSLIFIVYPQAIATMSYSPVWA
VIFFVMLITLGIDSTFSGIEALITGFCDEYPRILLKRREIFVGVVITIYYFGSLPGVTYM
LFGITVYNVNFSPLEVGEYVYPTWSVYFGWFLRLLSIMA
>ppa_glt-1
MASHPFFKWLRKNLLLVLTVSSVLLGIALGFALRSSHLSPQTVVLISFPGEILMRILKMM
ILPLIASSLISGLAQLDAKQSGKMGSLAIIYYISTTVIAVITGIFLVLLIHPGDPTIKEE
IGQGTEGKRNMFPENIVAATFQQVQTKYVNVTPVITPAHREKNFSLAPYIKATVEPAPGM
NVLTVSTLDTLLDLISYDNLPMTYNCLEENLGVDRRVTRFVLPVGATINMDGTALYEAVA
AIFIAQMNGINLSFGQVVTVSLTSTLASIGAASVPSAGLVTMLLVLTAVGLPVKDVSLIV
AVDWLLDRIRTSINVLGDAFGAGIVYHYVKNDLDEHDAEHTRKKLEGGHGDGES
>ppa_ser-1
YTDNAVALLLLPFLCATGLIGNAFVFRVCVAIGTERRLQNVTNYFLFSLALADLLVCVVV
MPIAGKWMWSNGLCLLYVYADVFLCSASIVHMSVISLDRYLGISQPLKTRNRTKTAIFIK
```

IILVWIITILISCPIVVLGLIDHRNVLNDQQICAVHNRTYMIYGSTFAFLIPFIIMTVTY
FKTTALLNKQAMLLQQGNSNGAKNGLRRAAPPRKLGYSNEKSRRSSGGTSTPTISAVNHA
KWSSSTATATSLHNINGAIQTPVRSKRSLPPSIWQKSNGTTTPAKDTLSDDLDLRPRRQK
PSRLQRWTTRTSSYLSLIVSRVQRKSSYATSVELLSEHKATRVLAVVFICFFFCWTPFFI
ANFIFGFCGQSCEPPVAISTLFLWLGYVSSTINPLIYTVFNKRFRKVAYIVMRLSESSVV
DVSAREVRITPMWDRTPIGRGHTQ
>ppa_ser-2
MTVVGNTLVVVAVFSYRPLKKVQEVQNYLLVSLAASDLAVAIFVMPLHVVKFLADGKWLL
GIWVCQFFTTSDILLCTSSILNLCAIAIDRYWAIHDPLNYAQKRTPKFVCGVIVILFVLY
SASGSFFVPLLVMVVVYVKIFISARQRIRTNRGRSALMRINHPPPEVRDGPEVSRKPLST
KNGSLGRKCERQPLVEADTASAQPLRQESSKLVDEDSSGKTHSEDTITKDMKYAHSVTVK
KLDKDKDEENPANVLRKREKISVAKEKRAAKTIAVIIFVFSFCWLPFFCAYVIMPFCESC
TLHPKVLQAFTWLGYINSSLNPFLYGILNLEFRQDSVSEVRT
>ppa_ser-3
MDSSEVDTRVFDISGGGGLDHYTTQAPTNSSVGPQCSFYEWSRLPQTAGLLRIFSTISVL
TVLVVIVVLGNSLVIAAVLLRRRLRSATGLLILSLGVADLLVGTVILPFSIANEVLAGYW
IFGETWCTMKSRKANNGTAPPPYWFRYEISTLPGAVAAKDSPGRTRFSNKRAHFNIWLTM
DIWMCTASIYNLVAISIDRYIAIIKPLNYPMLVTKFRARCIVAFVWVLSFIICTPSFILA
STDKPNSDSTDPAEGCKCTPSHAGYAYIIFSASASFYLPMAVVIFVYARIYIAARNATKS
VYSGMMQVSANANKNAKSYLMSHPSALGGNREAVPMLRVHRGSSVAAPRASDERTRRSVT
PLVRAPGVPEGSPAPPVPRCSPNGNARSNSCGMQSTPMKKIVEREKASTDADSSSPHSSP
TRTITAPLKTLEVAAGNGAAAACNNLPPWIADPKPDKRRLLDRSILKKGPLGRLLKKSPK
KKAGCAYEKRLSLEIKAAKTVAIVTGCFIFCWLGFAILYGFQIKTSEVLWSILFWLGYLN
SALNPVIYTVFNREFRICFKRLLTCHRVNQPSTKATYTNNNSYNSTVRGVSTLNNKSSGG
GGGVYTQAPPPLPVHSSPAHLYQYDSQGTATVKIASTDSKS
>ppa_ser-4
SVCLERELRARPQFYLIFSLAVADLVVGLQVGLFVSPLYTWSTVRGQWTFGVLVCDMWIT
VDVLMCTASILHLVAIALDRRYWSITDICYVQNRGSRRVIGMLACIWIISLLVTLSVSLA
PFANWKDDKFNYRIEVERHCLISQIISYQVRYSSSIQVGVSIILLLIQFQIFSTCCAFYF
PLTLIIVVYWKIMRAAKKRFKRERDRRTIHRTTIDAMKKKTKKTKESIEMKRERKAWRTL
AIITGTFVACWTPFFILSLIRPTLGASVVPAWLDQFASVLGYLNSALNPIIYTVFSQDFR
TAFKKILKRLCFIHDY
>ppa_ser-5
MKRAMSPTEDVSWKNKVAGITLVSGMLSLPASIIILMTIFGNLLVLCFKARVGRTNTTLL
VWNLGLTDFLVGVIVLPLGAFHLINQQWIFGDFLCHLWVAADVTFCTCSVVTICVISVDR
YMAVTRPLRYKSIVTKSRVILVMIIIWSFSISIPLATWKWNDLSIRSDNGNSKCFAGDQI
RYLAHSVVLAFFLPASVTLSLYWRIYNLARKRQKALDRGFLMILGHNMNFLTNTISQQTT
LRVHFGRKNGMVEHQRRVLRTHERIAKTLGVVSCSFLFCWLPFFSLYLTNFKCSCIPPMA
IDVASWLGYCNSMLNPIIYSFTVKEFKKSAQRMMLPLWTLAHRLLPFIRPPPDGLNLKMS
RHGKNRKSRPHSFVELGKNKSGVLTSRVTRQRRQTEPAVFASLDVQKRLGKVAAKDAETP
MPTTIIEEDDEVCYSEVNSLTSYSSSFSESRFVSPRKKSSTFTRMPTLTERKSLELTTSN
GVSASSGGPSEHSIHMHYDTIEADTKFVAWNCPNGTEYTMDDIAKPFDSCVIMTHATLTI
DSVNDTYWNVVYTSSDTTET
>ppa_ser-6
VWISACLGLVDLIVVCGNIFVLYLLASQPSLRSSTNKEHHERWLYGEIFCRAWLSLDVFL
STASIYNLLAISVDRRYMAVRQPIRYPIISSKRCSPLQFPDPFILASASASFLIPAVLMV
RGRGLAPSLRTELRVARTTSVVVAAFVICWFPFMTIYLLQFLFQVYDLCPTGPCVPSGLF
TFTFWLGYANSALNPILYAAFSRDFRAAFRRV
>ppa_ser-7
TMAGNAMVCLAVLLVRTLKQPPNFLLVSLAVADFFVGCIVMPLGLVSDLFNTWILPSFLC
GVTVLDLALCTASIVNLCMISVDRNIFRYLAISRPLRYSAQRTTKRILYYIAVVWIAAAI
VSLSSITFLIMKEETVKTEETKETCDVQVPQDMYYQIGATIIAFYAPTIIMVVIVYVKIW
RAAKRLAMQDKILGVESYVNKVTSSPLPDKRVSSSSQTSEGERKLLHRPSQAQKTLGVMM
GIYIVCWLPFFVRALYCALRGVHPDTKFDLVVLWLGYSNSMLNPMIYCKYNKEFRVPFRE
MLCCRFSTLQNVMRHEEFNNKFG
>ppa_glr-7
MPNNFDNYIAVVQPSSVFQYSIVDIFPQLNISTQLTVLSDDIYNVAYDSWKASFDSLGVP
VRYMQMATNPSRIRAQLNALRNQAKTIVFVAKTENIEKFIVEAQSNIENEDFKMFVMTKD
IRQYQEFISKNEINKDMDYMIQSYEEMDISLIYDLMNSTFQYLMKVNSTYSDISTFVCGR
NPTANTTLPLSPFISSRPVLEYGKMMGNWTVETGVLTYYESLTQSPRDITIYRIATVVQP
PFVQRTPDPLKGPNFEGYCKDLIDLIKENIGNFTYILYEVEDGTFGTMDDNGNWNGLMGA
LVSGSADIALAPLSVMAERENDVDFTVPYYDLVGTTILMKKNDVEYSLFKFMKVLEWQVW

LCIVAAYVITSLLLWVFDRFSPYSYSNNKERYKDDLEKREFSLKECLWFCMTSLTPQGGG
EAPKNISGRIVAATWWLFGFIIIASYTANLAAFLTVSRLEQPISSLDDLAKQYKIGYAPM
KGSASETYFRRMAEIEEQFYNIWKDMSLNESMTPRERSRLAVWDYPVSDKFTNMWRYMTE
SGLPEDLDDAINRVLTTPDGFAFIGDATEIRYAELTSCNLQAVGSEFSRKPYAIAVQTGH
PLKDEISSAILLLLNQRRLEALKEKWWNDNPKRKVCPDQTDESDGISIQNIGGVFIVILA
GIVLSIVTLTFEYFYYRRATPVTKDVVTSKEVIPKIKEHQSPPVSRDEENKEKSRQGSVI
HRRPHSSSNSPDPRSLPPQGDAVYENAAFDYPKRR
>ppa_glr-1
MRCLILLLYLFQPSLPYPNKIPIKSFTISIENGHLVEKAVKYSRDEMNRRESPPFRLSLD
HIEIKKEPTEAYDMITTVCQELKEGAMGIISLKDGRGYDSLKGLSENLEIPLISLNPPSF
PPEYPSRILIDTTSTSRLQKLLSTIRAAQFNQGNYHYVIVNFKHLEEKKDESEISLDHYT
DITARVALAHDTVIAAFHGFLRALSQNDSIFSKSFRHGKLYNRGYRGIYCDPSTDRENPA
RPYSSFEHGDMIGAALHGALSWVQGKGFILNQTISDHTRKNHEDLNNRIVKAGSKRPDGS
WDGLVGDLLNGDIDVALASLTINQDRERVVDFSKPFMTTGISIMIKKPDKQEFSVFSFMQ
PLSTEIWMYIIFAYVGVSVVIFLVSRFSPYEWRVEETIRGGFTISNDFSVYNCLWFTLAA
FMQQGTDILPRSISGRIASSAWWFFTMIIVSSYTANLAAFLTLEKMQAPIESVEDLAKQT
KIKYGIQQGGSTAQFFRHSSVQIYQRMWRYMESQVPSVFVSSYAEGIDRVRNQKGRYAFL
LEATANEYENTRRPCDTMKVGANLNSIGYGVATPFGSDLKDPINLAILALQERGELKKLE
NKWWYDRGQCDQGMNQDGSSASLNLSKVAGIFYILMGGMIASMIAALGEFLYRSRIEARK
ADPQLCGNFARNLKTALSSQLQLSMKGGATAHPGTNSHEALKRQKIITYDPSEIINNKEL
>ppa_mod-1
MEQFGVTCPVKVELVWMDIPITFIHYIQLPDFDMVEFKVQKRNLLYPNGQWDQLEVEFTF
KRRYGFYIIQAYVPTYLTIIVSWVSFCMEPKALPARTTVGISSLLALTFQFGNILKNLPR
VSYVKAMDVWMLGCISFVFGTMLELAFVCYITRCQNSSRWGKRSERRPAVAATRSMRNGG
VTNRRQLHLDDETTTIPMMNGNINGNGTVNGNGSCRDGTMTARSIHEVIHPTSSPSPIHF
HMSTFDSSTPLSIDQLHSNGVEISSHYHPHPSRSSPTPGCFMRFHPESVDKFSIVCFPLA
FTLFNLIYWWHYLSKSFEENFTMPADAFHSPPPPS
>ppa_ocr-3
MSFNSSDTRGSLYGLVDEHASGILVSWIKYARATGDYGILDEYCETAITLVIDDSAHNTK
DDSKGKSGPNILDDINQDKLEQADFLKGNMGENLLHICLLHNTVEQNELAKFLVQKFPRL
INDIFISEDYYGLSPLHQAIVNEDMEMMYFLLKNHADVHQRCYGAFFCSEDQKGSRTDSL
EHEWVDLNPNTRYTGQMYWGEFPLSFAACTNQQDAFRLLKAFKADSNKQDTNGNTAMHLA
VIHDLPEMFTLINQLGANLHVRNNQSLTPLALAAKLANKRNMYDLILRLEMDVVWRYGDT
VCEVFSLEDIDTIRQDNGDLNPSSVLANIVYGDKSSHLDFFDGLIEDILEKKWEVFAKKR
MFLSLFGYLWFLLMLSISFISRDLDSEKEEAASSSNTMSNSTNFANASLMSALDADPFEN
YTLLESILFMDDVFRIKIPWQSTQELPTQCHLWDHRRRKDKIRLVAEILTLVTVILRTVR
DAIDIQRSGYRRWFSVMLAFPEKVLHKVAQLSIFLMVPIRFMCALSPAVLLFENIMIIAV
VIMSSLHFFFYCRGLKFVGPFVLMVYKIIAGDMLRFFVIYVVFITAFAQAFFLIFQSCER
AELDFLAHNPHREREFENILFNSFETMMRMFIMSVGEFGMFYKNLNDCKSNLAGLGKEFS
LICARSSKEERRRRAREREEGERHTQIRLRPLPSRLAFPSSSPVFFTLYELIVTIMLLNL
LIAMMTRTYEKIAEAQKEWKRQWAQVILMLEQSLSAQERLLALYSYSRPLKSDKRNRAFI
VKEKRPSGNDSESASAYLELFDGVTREHISQEELRHLEIMPRKKLSIARLTNSPIIRIND
SSLPSLRDA
>ppa_dbl-1
MKSGTAERSLALADVANVSVGLDSISVSPPPPPLRSSSFILSSTPFLYLFLLHTLPFLVV
ASSTPGTAAPPLQLVVLAKLGLSTPPSLSSPQLPVPDHFLEEYRKAEEGMLDQDSIRLFY
PQELLDLNSGLLLSYNLSIDAQQALKERVVEARVKLRVRGGRGGSLRVYRVEDMDRLKVE
RLLDSVEIARDGEEERLVEMDVTETVPYTNQQHVVRLLVVLPEGCALVDSPANSLSSLSQ
SRAASASMVVSYVVRDGDDLQEENEHPKRRKRFSDEEKRRKELRKERRRNNRRHRTGGQK
GLCHRKSMYVDFEDLGWLDWILAPTGYDAYQCSGGCPFPFPAALNASNHAIIQGLLHNYN
DEASIYLTLIPGWFPSENNPARPAPAGPKRPHAFLWLVPEPCCVPTTMAPLSILYRDLDN
RITIKNYPDMIAVECGCR
>ppa_unc-129
MEGIQSESIDKFKIKRVFESLLELDSPGEEIGGRLPIMAADYMKNLYMEAAESGMKGTVR
TVAPLIDRWDGEEVLVFPLSHLQIGHRLIRAELHMLGGRPDKMGRALRVEGLVGERSSSI
HIVDRIQLKLSSVSTNAVLWNSVHPILYNFANSSNLVIQLSRWDGRRMRPLSSFISRHSP
FLTIYTRSEDEDIVMEKREGGRNKRSIDSGSYFAYESEDSSVLRKGQMEYIQKGPSMLNT
RVKNTKFTRRRYEKMKNKQPFWGFGEKEEEKEVSARILKIGNDDEQKERMGERTLEMTTA
VNEGDVDVVLLHPEETKNTVCGKQPLRVDFAEIGWSNWIISPSYFDAGFCGGECPFPLPK
EARASNHALVQSALSSSFSGLPSVCCAPDRMESLTILYYDQNKAIVLKSFPRMIVESCGC
I
>ppa_sma-3

MNTLFTWSNPQVKKLLGWRTGSEEEKWAEKAVESLVKKLKKKKSGPGSLEDLEMALANPG
SLSNLIDHVFGDTPRFRRYATSLAIRHVFGDTPRLRRYATSSAIRHVFGDTPRLRRYATS
SAIHHVFGDTPRLRRYATSSAIRHVFGDTPRLRRYATSSAIHHVFGDTPRLCRYATSSAI
HHVFVDTPRLRRYTTSSAIRHELRSIPECLYPYEQKAEHICINPYHYQKIENPNKPFLQS
ASTIGGHATSPYSDSYASGSSGLHPGSIVSSIAHADPIAHGQLLFHRVTIQYLFLIPDRR
LALFDAIRGRRPNGHGESAGGDSVSGSFEI
>ppa_sma-2
MLRMENITKHLNWKQGDEDENWAKKAIDNLMKKLQKHDKSSVMRLEQALKALPHVIYCRV
YRWPDLQSHHELKAIETCMFCYESGQKEICINPYHYSRIDGVGVLPPVLVPRYSERPPQE
DIPPVFMRLQRMEAESSSGMPDNVDVSSGRMKVEGPVKEDLDANYNIPAHLPIVPVAYED
QDYWGTISYYEMNNRVGVQIRMKSDTIKIDGFTDPLNNPDKISLGIFSNVNRNATIENTR
RHIGNGVKLTYVRSLGTLYAECLSDSAIFVQSRCCNYARGFHMTTVCKLTNKTSLKIFDA
TMFRRLLSTSASMGFDASYELTKMSIIRLSFVKGWGAEYQRQDVTSTPCWIEIHLHAPLM
WLDRVLTQMTPPNAISSMSQ*
>ppa_daf-8
MYTEIKELVNYLVPFLFNKIPRRKVITFAEQLANTLLFRLREKWNDCNADEISRVVLIRL
RVATQTGVKEQTDRDVILSAKAVGIDVDEMFRFLPGQSFLFPSHPSKLFHSEGIAIHIGP
GEVLYQMTNGCTNASSTHLIWSGNPDCDREYRPLPAVLTLPAEETEEGTVATPDNYVPLK
LAPVNCEKDLTKVDTLFVALSRHPSEYTVEQFSVTRFGSHRARPDHEVMKRIQRQAAFEA
LTKSPIVDGGEDSILSDNSSLLSSSSRTVIPPPPPPPPPQTPSPMDIIRSMSVDQRQMVM
RLLSDARNGQNNVFSGWNYLCPLRMFSWFNQFGKTDDFTPLLAHSLSIFDDRKFVDKAVK
HFSQRAVNSSTIENLNVAVNTKSKTSACVMVKRASEKNRINSLDKGNTHLLSCRIFRFGW
LLSVNDLKSTPNCPNGFSKRSEEYCINPYHYEPPKAKVSGVTVQKDRFPFPDTGFAEIPD
DDFIPNHTLSPDNPMIVRVNDNEDEEMKSPDSNFSEDIVMEDATEITEDPKRPGLFTVPF
EEMSCWMKLCYGELETTRTNILEVHGHQFTIDSSTNPTTDERLCLGYFTGMETVRDKKLE
AIRRAIGKGVRMYHTGGEIFLQCLSDSPIFVQCPSANRRYGMPSSSVVKVDPKTTLKIVN
YAEFANDLAAAVERGYESVYALTKLCGLRVSFVKGWGHEYSRKSIKQTGCWFTASFPAPA
KWIEKVIQQMGAPALFCGSVT*
>ppa_sma-4
MQQLHQHPMFGHPGPMPPFLNYDSMGLMPAHHLPLPPPPPMLPPPHSLPMHQPPLSAATA
AAALFPGPQLTLHHHPSTSMSQQQHMQQLQQQQQTTAAADAAAIHNAYSASASDSSSQIT
TVLMAFRQGGEETEFVRKAIESLVKKLKDKRLELEALIQAVTSNGKTPTCCVTIQRSLDG
RLQVAGRKGVPHVVYARIWRWPNVAKTELSKLPMCRVMPDNQDFICINPYHYERVVSNAG
ATHEQQPVSKMPVLQQQLAATATPPVYGLQLPFDGRGDEWPSSQITLTSAAALQADIRMA
QQLQQQSGETAEFRGSMFPAVEPPVMHRAHSIDLNRVIIPPAQHYPNHWCTINYFEHDTQ
FGESFKVRREMMSVRVDGGLDPQGERNSRFCVGAISNVHRAGPVEVVRQHIGMGVVLKQH
QDGRVTMEVRSNKAVFVRAPYMDYMKHTPYTTTIHKVQQCDGEITIFDLKWSYFEMCEQT
ASAKEAVVAQARAVAGLPVNHPGMQQMSDMVAGSGVDEMKRMFCSVAMSFVKGFGGQYNR
KNIKDCPCWIEMCFL*
>ppa_F37D6.6
MDCPLWCLLSDRRRHVEHLWRRRLLVTADERRARRRFLRLMRGFDTEDVDVLRRAIESEG
RDARQCAPGPPMEEREEESRGEDSGLIPQIDRPMSMPYLCCKLWRWRELQVDAALHSLDS
LPWCRFGRVTINNATVSCCNPYHYALWIRADSNDELPITSNGQILPSGETKVSSPVKEKD
EDMLLSDGIGLSGPPPLPPPPPPPLEEKEGMEAAIGLLANSVHDAQWDTRNEVSFALIRE
DVWLYNQEKEDESVPSTSSPRHSIHRCVSGFSFNSS*
>ppa_nfi-1
MWDTPIKMDHLEIDVGSVADQISPASLGIGAHNLSNEWAYSSTNSPLGVDEDLHPFVEEV
LPFVKDFAFTWFNLQALKRKYFKRHNCRISIEEERNIRNELEAEKVDVKQKWAARLLSKL
RKDITPTHRDLFVAAIKGQRAGICVVSNADQKGKMRRIDCLRQADKVWRLDLVMVILFKG
IPLESTDGERLEKCSECRNPALCINPYHVSITVRELDLFLANYIHTEDREALKGESCFRI
DISELQHIRRLDRMTMGKDSTWPILMKARLYEYFHFRRGVQWYGRTRNMGNSSLSPPHLF
PSNLINRITKMSDHHVTSPSTSTASAASSRVSTKATSSEEKRYKPSTSRSTHSTSGSPVF
LPKKDPQEGSVQCIRNEESHEEDTQ*
>ppa_C34E11.2
MGNMMRPDQVEEVLFKLSKGTLDDAIWGKIAIMEKNHRVAKVYLRNPTVIVDGSEEDFDG
KTLGFNAFANDRRDEQTTEIKQKIKEGVILKMDDHGNIKGMARGTTPIYAQGWREPKMNC
LSEKLVKMQGKITSDERSYKIFDMKRWKIAVERFAEGEGEMRSLLHRACIRVSLAKDGID
LTRTPCWFTIVNLVALDVLVSRCPHVLTGVSPSRPLPVVAQSLPSPAAVTAPVYHPPPPP
PPAHSSPSIDPTAQLPSIVAAVAAQMNQNLNTQLLNSEQLAQIASAVAAAAQNKDGKKSP
ARTKKDRRRNYRKDIDSDDTSEDSAHRSQSSSSNNKDAWKEMQLCKGPEQHHQTASHVEP
DSSNKEKTCIEDKSDAMQISLYKAKKKDAGSDSGGHVSKPPVEEKQ*
>ppa_tax-4

MLLQGTNAVASRSSPLAANGGASNGGIDENHPEDERNGICSKLFCRRSNRVGDSYLPSNE
SGEQPSGTAAPMQTISEPAPEAPRIDKYILDKSSNLYYRWIVIVTLAFVYNLIFNIARTV
FFDDLHFGYASIAWMFFDILTDGVYIADIVMKTRTGFLEQGLLVRDSRRIREQYMRSVEF
RWDVLSMIPADYIFNFVRKKSSYPNVFRVCCVIIGINDTLVRRYVYSFYWSTLTLTTIGE
VPGPVQNCEFVFVTCDLMCGVLIFATIVGNVGSMISNMSAARAEFQNKMDGVKQYMALRK
VSPNLEDRVIKWFDYLWSNKQSLNDESVLKVLPDKLQAEIAMHVHFETLRKDCESGLLAE
LVLKLQLQVFSPGDYICRKGDIGREMYIVKRGKLQVVSSEAEDATVFATLQEGSVFGELS
ILNIRGSKNGNRRTANVRSVGYTDLFVLNKNDLWIALKEYPDARRMLLLKGRELLRKDNL
LDDDAPDEQASPEEIVDDLLQSINVLQTRVARLMAEKTSTEAKLNTRIEILERELVKYKK
KRAAKQSEPRFARSHTLAADFDPHDLITPDRPGTSYMEPTDPHSKKNN
>ppa_tax-2
MSSRVLIDNSNNNNNNNKNNPILSSRLSFLVQDRLHLLVSEVKRRTSDVRETLLQEAEEE
EISSVTAKPLERQSSLNSLIGIDHELEDLDEKGEGRKPPIIFKRTVNPNSRLHMAWLSLQ
TFAFLYNAIVIPLRSSYPYQTKVEDRTIQNLIYWLFLDYFFDLVYLADMLIWKPRKQFMK
GGMPINSIRETSFKYLKSCEFKVDVVSILPTDLFYIWLGPYPIFRINRLLHFKAFGQLFD
MLDNSFSNPYAIRVARTLSYMIYIIHVNSCVYYVLSALQAFGQIAYKHNGKYYLNKWVYN
NQGNSYVRCFYFTAAVATSTGNNPAPTNVIEYIYMTFSWMMGVFVFALLLGQIRDIVSNA
NRNQEEYRRKMDAALYECQRLKLPPAVTARVRSWFIYTWEQHKTLDEKRLIEKLPLKLQT
DLALSVHYSTLSKVQLFQDCERALLRDLVLKLRAVIFLPGDMVCSKGDVGKEMYIVNAGV
LQVVGGENNQTVFAELGQGKVFGEISLLAIGGNNRRTASIRAKGYATLFALSKQDLQDVI
KYYPQAQAILKKKAAEMLKKDKKAEDSKITVAPEPKMLKVVAQILPEQSETTQILKKALK
DRRPSMLLRPDGETPDFSDLSDISDVEEEGEE
>ppa_glb-5
MASHPMYHEYRVHQLVQETLDTVMDAASSSIDSEDGASIKEDLRSDDTDRCDSDLEADET
HLARAHWILLHKMNKQGTVIQSTFEHLMTEFKHTRPIWQFGRNIDENVKDWNKELHEDFY
FRHHCASVQAAITMIMENKDDIVSLTRVLNEVGAHHFFYDAYEPHLILFEDAMITAMKKV
LKGVEELDEETERSWRVLLQLTRKHLIEGISIQRNGYLKQAITPQEHTEIMEAWSRVEEF
GLEEAGVKLCETAFETYKSLLSQYELSLPIPAVPGSNSDVFRQFSHMTMAVSPRYGFASL
PEKLSDYAITCMILDVCPTLVRKAFMEGKERMEDN
>ppa_npr-1
VFYEEYPDPSSSIYSIVPFALFYSTIFVLGLAGNFAIIYVTLKHRTLGALYVTVILWSLS
IIVTIPYAVYMYVETYEVRRICGCFCTEKWPNPTSRRVYTMVVMIGQFVLPFIVMAFCYA
TIFSRLRNRAKVRMNISQTMILDYDGPTILIDQPVLLSTTQSTQQCDVFYEEYPDPSSSI
YSIVPFALFYSTIFVLGLAGNFAIIYVTLKHRTLQTVQNMFILNLAASDIIVCLLSLPIT
PVTNIYKNWFFGSVLCRLIPWVQGVSIFICTFSLGAIAVDRYILVVHPHTPPLSKQGALY
VTVILWSLSIIVTIPYAVYMYVETYEGICGCFCTEKWPNPTSRRVYTMVVMIGQFVLPFI
VMAFCYATIFSRLRNRAKVKLRKMDACSHALESAQPSVSPLPNKNGTGGTELRTQLIDRN
GKGGGRHRILAQTRRTTMILASMVVMFGLTWLPHNVVSIIIEYDDSRTIFSWYDHDVSYL
INLFTHSIAMMNNIANPVLYAWLNPTFRQLVIETCFGRKPRRNVAVEQKFVRSMVAKSEC
TTRLTSRAQSPDRKEMANGGELDC
>ppa_hsf-1
MAYQGHFKFTHVKVMAYSAPKQEQMSRQDDQVAIMGGESVQVTREDEKIPLFLIKLWNIV
EDPSYQKIVHWDDSGYSFHIVDPYSFCRNVLPHYFKHNNLNSLIRQLNMYGFRKMTPLDR
SGLTRAESDQDHLEFSHPYFVRDHPELLINIKRKSSNRNENNSSGQVSQNNLGAVSEELR
TLRERQRHMDAKMANLVKENENMWEQISHMRAQHDTQRKVVEKLVQFLVALVGPGQKPRI
PRKRGMLALDEAPLKRSRSSSSQYSSGQVNVLSDVLDNLQKQLNDGNLRRTEGPIIADIT
EELSPTLSNSPPPSTSFIRPPSQYADICGSSMINGDEMGEASTSSALLNDTWENDKTLNR
TVTPTSPIFSINPSIDFQWGNDLQEYLNGVDQSIDSCRDILGRDNYQWDLDGSDLDVDRW
ITDDNGGQLALEGKNW
>ppa_hsp-3
MKTIFFLGLFALAALSVYAEEEEKKQEKYGTIIGIDLGTTYSCVGVFKNGRVEIIANDQG
NRITPSYVAFTPTGERLIGDAAKNQLTINPENTIFDAKRLIGREYTEKTVQEDIKLWPFK
VLDKSNKPHVKVSIGKDQKEFSPEEVSAMVLTKMKEIAEAYLGKEVKHAVVTVPAYFNDA
QRQVRGNGECAPVHERKACTPPPIGNIHHTSLVNDLQATKDAGTIAGLNVVRIINEPTAA
AIAYGLDKKDGERNILVFDLGGGTFDVSMLTIDNGVFELINNLVMLEVLSTNGDTHLGGE
DFDQRVMEYFIKLYKKKTGKDLRKDNRAVQKLRREVEKAKRALSSQHQTKVEVESILDGE
DFSETLTRAKFEELNMDLFRATMKPVQKVLEDADLKKDDVHEIVLVGGSTRIPKVQQLIK
EYFNGKEPSRGINPDEAVAFGAAVQAGVISGEENTGDIVLLDVNPLTMGIETVGGVMTKI
IPRNTVIPTKKSQIFSTAADNQPTVTIQVFEGERPMTKDNHQLGKFDLTGVPPAPRGVPQ
IEVTFEIDVNGILHVTAEDKGTGNKNKITITNDQNRLSPEDIERMLNDAEKFADADKKVK
ETVEARNELESYAYSLKNQIGDKEKLGGKLDEDDKKTIEEAVDAAIAWLESNKEASVEDL
QEQKKDLEGKVQPIVSKLYKDGGEGGEGAPPTEDKDEL

>ppa_hif-1
MPHGKKSNMDRRRETSRFAARDRRAKESDIYDDLKDVVPLVEEPTITHVDRIAMLRVAST
VCRFRKNVGNAILLAPDSSKLLRRSLQREVQDASFWSEDCLSGCLDGFILIADSDGVILY
VTESVSIFLGLTQTDFAGRHLKDFIAPEDYKDYLAATQELTATSVDEDLKGSRACVLRFK
TVISPRGRNLNLKSAIMKPVAFNIRVIVSNAGHCHLMQAITNPAGQGTISSSANALTKQG
ETQSGQFMTRHTCDMRLSYVSEQLHALLKTDSRSLMGASYYDLIHPADAERVRSSVAELL
VKGHTRTPYYRLITGMLRIYGKNILSPLLQVSLLTSGQGTVAWIMTEGQTVSHTTRGQKG
QYIIGIHYVLGVQSEEECDRGATRGISAGLAVRIKQEPDDREYLARQPEILDCIEFPSLL
EDPGEFEPVPQRNRRVGAFGNNITNGFNAEHNDSAHKKKRQHLMGTEDEDMLSARMKVVD
VIGIPPLAKRTRQDRSPSCCRHCCCKTPIDERKDCEHLARRASKINCNGFFVSNQKEMEK
RKLRAERRERGEECGEKEEEDETPFLEYSLVAPVLRSTHSLILHTDERPSGLLVRESKEE
SSEESSDEEEEACCKARLCSRSTSPITDANSPFPLRRMRCRRRSPVPATSPRPRNRRAAL
ARRPPLAPILLSQLQALNVNYEPRNFAPAAPTRMDGSGMAFQRTILSHCPTVQDEVTHAG
TPRAKLSITDVLSTLPPAGFSAATPLVYGSGRAPRVINNTSLVSSGYGSTANSCSPSSSS
STASTPMAPSIPIPSSSSLRPTPYGSLSSSSKMLGDDSFDMNAPFIDPCYDIPLGTGGLP
QDLVSLLEIPDWVEPEELELPPRNARMNDYSSISQGGLFGEYCSHPLPLTTNEHRELTIY
IPSLQMIGIHLPSSMVACNLDGVSIYYRLPYHELLYVTQIVIDFVVVGIR
>ppa_T14G8.3
MQERNSIEEVTANDEDDHEVNSGIIIDCCGNWSSRINLYLDAALAAMSIDFGSQYIKLAI
VKTGVPMEIVLNKESRRKTPNTLGIKNGERFFADAAAQLALKQPKSTYPYLLELIGKKDA
DPAIARYKLQFPFNEFTTNNRGVALFKNGESEYDIETLLAMILWSAKKTTEAYAEQSVKD
VVITVPAFFNQAERRAIALAADIAGLNLLQILNSGSAAALNYGVFRRKEISEQSTTMLIY
DMGAVKTDATIVEYQLVEDKDKMKNPVVRTLGYGYDRSLGGLEMTLRLQKHLEQTFRKEK
KTPTDIATNPRSMAKLHKEAERVKQILSANSHTFAQIENVHEDTDFRTEVNREQFEALIE
DLDDRIRKPITDALVMADMDLSKIDQIVLMGAGTRIPRVKAVLSSAVGGRELGNFLNTDE
AIAMGAVYQSAKLSKSFKVLTFDIQEPVLFPVKIDFESQDEQGNKKAASKVLFGHKTFYP
ANKKIVTLNSYSDDFTIALNYADLHFDEIQATEFGSRNLSSISVEGVKVKMEEAMSGEGS
VFKGFKVHVRIDDSGIVSLDKVELNVEQPPPPKTAEEIAEEEAALAEEEAKKKEEEEKKK
NDQEKKEDDQEKKEGDDDKKEDTKDDMKEEKKEEKKVEEKKEKKKEEPKPKKIRVKMTVK
SEHTDLTLPNEEEIVAAKKILAEFESIEAAKQAREEAQNGLEATLYDLNDKLETKEGVIE
FMTQEEQEKLQAQVSLLRTWMEDEAGMDTTTEEFTDKKKSVDDITEAGRKRMDEYRAFPQ
AKADLDALFNSTEMFLTLAANMTKKDETDEEGIFNDTEITTLRTKYEEIQQWFEEKVTAQ
VNAAKSDDPSVTQDEIQEKGVQLQREVKYLYNKMASYKPKIYINCYNCFRSRRKRKRKSL
LLKKRSLKMKRQLRKPKRRKRQKLLNLLTVICKREYC
>ppa_C17G1.7
MVYLVAAKLEYLNPACSVKDRIGTAMVKDAEDRGLIKAGVTTLIEPTSGNTGIALAFVAA
AKGYRLILTMPASMSSERVRILLKAYGAELVLTDPALGMKGSIARAQELKEKIPNSYILQ
QFENSANPRVHYLTTGPEIWQQTEGKVDACVFGVGTGGTITGVGTFLREKNPNIKMVAVE
PEEAAFLSGLPTGPHKIQGLGAGFRPGVLNTDVYDEIIRVHSDEAILMAKRLAFEEGLLC
GISSGANVCAAMALASRPEMKDKLIVTVLPSFGERYLSSTLYADTRDAALAMTIETLEQN
VAELRLHENFEIGYGNGLNSGISFNLHTSFYTSIRRS
>ppa_egl-9
MSYCCKCKKWRTEAQLLLDKDHDCERERDTRDWSRHKVWCKVHSSRAHSARSSAESSLSA
ATAAASAASSGVPNSSTSTAAAAATLPATKRMESIRRIDSGHALDEQPSSSTGSIGGVKR
MAMTTSSSGSSQTSGHSSGTDCSLASMGSTAAAAAAAAAAAAAGNNDDDDPMAGMSTDQL
LAMLQQEQAAAAALSALPLPIDPTALLAATAANPEFLAQLTLMIAVQQQTTAAVAAASAA
PPPQPQPQALPAMPQLFPPLGPLSQQLGLSGFSMPPPPSAFPSVSTFDPVNVANMISALT
AQAKQHQQQLPQQQQQQPRFDLGAADPTPMMFVSSSSSSSSSVPPPRGSAPSTSAFAPPP
ARQSVVTSTFAQQQQQLQQVQQLQQQRPFAPPPPLPAVSSSSFSRSAFSSTSSLHSAASS
SSLRSVPTTVPNPFTYDTTPSSMQKIIDPKPSATVPAFLRTKAVAVRRGGKPAAVAAAAA
AAANGEALQRLLLQQQLQQLQQQQQQVRPSSSGSGGGAAASSTTSSGGAEGAAQKRRSPD
RPEGSRKRTTPSPKDIIKEERPWSYVNNDDIKNMPIDYADVMKQLQGPACTLAKVDKPPV
VEDDDDDDCQVIGEIINRRYRDHQQNLIYNFSVTEHVENMRSKNMIMTRHQATMLRIRYM
SEHVIRSLNEFGWAVVDNFLNSENTKNCAKEMEKLYEKGLFTAGQLVDNSDDEDFHHERN
EDDVKSVRSDYIFWYDGVDPRAEEAVSTRLLVSMLDALMCNFNGRIVDKEVGGRSRAMLA
IYPGGSTRYVKHVDNPNYDGRLVTCIYYCNPGWDLKQHFYLKYIKERQLDPAGGKHTAAA
IRLATLASHGGALRLFPETSDTPIDIDPQADRVVFFWSDRRNPHEVMPVHRHRFAVTIWY
MEKNERRAAQDRKMKRKAAAAAALGIKVEDLPTNRAQLARMLAMVQPQYGELLKLGSIPQ
THASTPHMANAAAAATANATALGLPPQA
>ppa_gcy-35
MTFSRWEYDSCSEKHREEDTVVVRPFDRLSFLPSSVEQLFGWIHESFRQLVNRKYGREVW
LKILELSRFEEGTESEISHYYNDDETLRLVNAMANVIGIPIEEVWEAYGGFLIQFTMETG

WDELLRAMAIDLEGFLDSLDSLHYFIDHVVYQTKLRGPSFRCEPQPDGTLLLHYYSKRSG
LYPIVKGVVREVARRIYDTEVVMKVQERKQEHLDAFVTEHVVFVIVQVKTSNTASAKAIT
SKISELPMAAITAGIGMFDISAEDFCLAYPYHICFDKDLLIEHVGAHIRRSYPQCQRQET
RVSDIFELIHPEMPLSYESILAFKNSLFVFKMKGSGDVVHEGNDAAEGKPVHVKGSMTLV
DGGKYLLYLCSVNVTTVRELIERKLHISDMQRHDGTRDLIMLNQSRMSQVELNRTLEETM
KNMKKMAAELEIEKQKTDTLLCELMPPSVAESLRQGKVADACEFTDCTLLFTDIVTFTNI
CAECTPYDVVTLLNDLYLRFDRLHEVYKVETIGDAYMIVGGVPDPCDNHAERVLNVSIGM
LMEGEGHESKLVGPTTTCLHPAVTTSKLVHSPITHKPIKMRIGVHCGPVVAGVVGMKMPR
YCLFGDSVNVANKMEACGVPCKIHVSEPAKKNGSATNTSFVFSTRGLTEIKGKGLMFTYF
LDRNDRRSVWELCSRPRSSEQTIDGYMELHDLGIYGEIEDTPQGSIKMKKNGKKKREPTA
MEDWLNRQRDSRGNRNEQRNHEMLHRACSNEGTNSRVYPKHF
>ppa_gcy-36
METHSIYPHFPSSFGFIHESVRQLMIRKYGEEFWQKVLARAGFEAGKENIVNHYYADSDT
YLLVDAVSVISKMPREQVWEMYGSFLIEYTMEIGWDDLIRSMSPDLKGFLDNLDSLHYFI
DHVVYKANLRGPSFRCEENADGSITLHYYTGRPGLYPIVKGVLREAARRVFKLDVSLTIT
GRTQRSVQMSTGERTEEHVIFLIKTAATSGKTDHDMFTAVMPSPTSGVCRLTAQDFVNTF
PYHFVIDQDCKFVQVGRELTNYVAPDLLTSGSSLIRAFEINRPQKMMLLKTGIHIMYLCS
PYVTSIPELLQFGMRLSNMPLHDATRDLILLNQQRLSDVEMNLQLEASNEQLELMAKDLE
KEKTKTDQLLSEMLPPSVAHQLKLGQSVDAREYDCATVMFSDVPNFQSIVPNCPPKDVVF
LLNELFTKFDRLVILQKAYKVETVGDSYMSVGGIPEDCENHCEIICHLALGMLFEATTVC
DPIQKTPLAIRAGIHSGPVVAGVVGAKMPRYCLFGDTVNTASRMESHSPLGRIHCSESAM
NCALKTGRFQFIPRGKVQIKGKGEMQTFYLVRSYKKSVWEIVERPRDEEVNTIDGYDEML
DMFANEDELGKIAKTRVCSLQPALAEKMNEVYKERPANFFNEKNCEAELKRILNEPCPKP
IAPNEPSSTSYSRATVIDAWIDDLRGKINAEYDTRIETTLVDFRKMTELIERLMSKDNRP
SMEEMKAKLEQINEENRKRSRSAEETAQLERVISVLRARISQIFKERREANNDADKALPP
GSLSISPTKASSIALQNAFRSPSPIKSPITSPIRPLAEAIEEANAVLGVEEEEMEETEEV
PEEIENEEAETEDEPKMEVDTPVASPLNRITSPIASPIVAHIPSPISSPIRSHITSPIAS
PIRSAVVSPIRSPIATSDEKRDKEKSVSPRTAAERAIGDGGLEEEMEEEDDEKSFWGSLY
RVGILDPQL
>ppa_sulp-8
MPTVFQSYKECAQSEERAATEGDGSTDPESLVEVKPRRRRPSIQKRDVLNAAKSFMPILD
WLPKYNIKENLHGDLIAGLTVGIMHVPQGDHILFRHTHATVFRKWEDSKKIVTGMAYASL
AAVPPIVGLYSSFFSSSFYTFFGTSRHISIGCFAVASMMVGAVQMQLMPATDVNGTTAAP
GPLGDVTSLELTSALTLAVGIVQFVMALSRLGFITAYLSDPLVSGFTTGAAVHVFMSQAN
KVFGVKMPRHSGMGMIIYMVRDIIKEIPNTNLMALAISVFGIVFLSIGRDYVNPYVKKYS
KIPVPLELILVILGTVFSVVMNLKEDYHIKIVDVIPRGFPVPSPPRITLILHLLSDAVPI
AIVCYIFIISMAKLFAKKHKYRIDSSQEMYACSFFSLLSSFFPVYPVGASLSRSAVCELS
GAKTLLYTVFSSALLLTVILFLGPLLEPLPMCILACIVIVSLKSLFMQVKELPRLWKISK
HDFAVWSIACLATVLCNVTQGLVIAVAFAIITVVLREQWPSFTDVPLSTIASSQKVPDFA
KVLKFDAPLHFANVTRFIDVLQGTFADERIPHTMNHFLNLVVIVIIHLPSPFQTLPSEKV
VVVDCAAISYIDSMGVDALKETYEDAKRAGVSLYFAGFNDSIIQMLNTIDFFNTVPLSAF
QSTVADAANQSI
>ppa_sulp-1
MDVISILGPSGLQRSMNQDEFDARFSYISPTEKSQQKDEKAKWRNCLSRCMKEDWIDHKS
IHDYLPALQWALKYGMKSDLPRDLAGSILLACLFLPQVTVTAVTVTVSAVTAGVLAVDNA
YAGIYALVFPQLIYPLFGSSRHSSIGALSFVSLLVQSSIANSGTNAAGLALVSALVHFLL
VILPLDPLIALIPDTLLSGFAAGLSLRLLGFFFSHAFTLNECTERILEVQLSSLPSCLLS
STGWLLLLASIVLLLLSIAHFLTLIRFTNSIPVTPPFQLAVMVASIALSYTLDLPGHGIS
VLGKGGLIRTRNDNEWNRNTQ
>ppa_sulp-7
MGGAGTIKTLESSSLVGTTRRRSMAGKIIKKSAAAVLSFLPILKWLPRYSIKKNLLNDVV
GGFTVGIMHVPQGIAYASLAGVRPVVGLYTSLLAPLFYMIFGTSRHISIGVFAVVSLMCG
ACNVRVTDEFFAESSSNYTDEELGEAKLDYALHVLSGLGFVVGIIQISMGVLRLDFLISF
LSDQVVTGFMLGASFHVFVAQLDKLLGVTLPRRGGIGKLFMTLSDVIQSLMALEINLYTT
VLSISAIIFLFSIKTFVDPLVKRVTPLPVPYDLFLLIIGTVISTIFDFNTTLSMKIIGKI
PTGLPSITPPDLTVIPHILGDAAAISVVILVVSISMGKVFSKKHKYEINVRQEFFALGLV
ESLGSFFPVWPSSTALARTLVYEAAGTKTQARTLKRKKKLATVFSSFLLLLAVLFFVGPFL
ELLPVRSGFAVFALLRDRRRTQGDVHEARRYPRTVEGVQDRCGKGWR
>ppa_cey-1
MNTQDIFVHQTAITNNNPKKYLRSLGDGEEVLFDVVEGSKGAEASNVTGPEGEPVQGSKY
ALDVEQVRTRRRTRGGRPRRPTRGEEGEGGNGGERREEESTERDGEEQGTGIRPSRLKGS
NRRRGGRGRGRGGRGRGGRGRGRGPRQGGESGGEQGDEVR

>ppa_kel-8
IAGQAVTVPAATFWRPTITMNDDHSYAAAPCFMTDRLRGQPGDRGSTFHSQMPSSFYASS
SSSFPPPDVQEIVDVHYPPVVLGSLSELREDEQLCDVELEAEGETILAHRVVLAASIPYF
RSMFVSSQMKETNQRRIVLCDISSSALRQLINYVYTSRLLISGENVQQLLFAASILQMDA
VSDHCQKYMSQWLTAQNCISLRQFAEQHNCSSLLLSTDAFAVEHFAEIRLMPDFLSIPFA
HIRDLISRSDLNVNHEQEVFETMLQWLGEETTRRDHLPDLLQHVRLTQLSCGYLQEVVMK
HPFIATEPSCMQQVAAAACSLAVGFSGAGMGSSSFNDAAMAASTSKSSENTGEYAASLPE
GETRERRTMEQCPSDILRPLSPAGGRVWPRKSVAGVIFCVGGRGTAGDPFRSVEAYDWRR
NRWFAIAEMTTQRRHVGVVSAHGKLYAIGGHDGANHLATAECYDPSERMWKQLAPMRTCR
RGIAVGALEDAIYAVGGLDDAACFQTVERYDIESNSWTNVAKMNIQRGGVGVAALSKFLF
AVGGNDGTSSLDSCERYDPHLDKWRLVAKMMNRRAGAGVCVMDGFLYAIGGFDDNAPLNT
CERYDMRKDEWTQLANMSCARGGVGVAAMGGLVYAIGGHDGMKYLNSVEAYDPVLNQWRP
VSSIKECRAGAGVAWADVRVANLLSPKHTHESGCAPYNGIAPCV
>ppa_unc-58
MDGEDGATVVTDTIKDDEDRTEPKSCPQQTVKYIKILTPHIILVSVLIGYLCLGAWILML
LETDTELQARSKKLVRLTNMMTNFTIESWQTLNDAQHGLYKVEEEEWTQRFKEYMLSVSE
VVDDRRPIRRELIRPDDLRNMHNKWTFPTSLLYVLTVLTTCGYGEVSVDTDFGKMFSVAF
ALVGIPLMFITAADIGKFLSETLLKFVSQWNRMTRKIKSFFCRSRYGRRKSMQSSNGNTD
TLDILGVDGAEDKLWFPIGAYVGCICLYCSMGSAMFINWERKWSFLHAFHFGFNLIVTVG
LGDIVVVDYVFLSLIVAFVIVGLSVVTMCVDLASTHLKAYFTRIHYFGRAKRFLGMSEEL
KEIVALLGAMRRKKGGKVTWNDVRDFLDNELRDRPFEPHELLMKLRFIDETSSGMSTIRH
NSFQSDFYRESEYIRRVNALRPEQPAYL
>ppa_che-1
CGKSFSQAANLTAHRRVHTGEKPFSCPICDRPFSQSSSLVTHKRTHSGERPYACGQCDKS
FTDSSTLTKHLRTHSHKPYSCNMCMMRFTQSGNLHRHMKTHK
>ppa_exp-2
MEEDINHPFQIIPTEGTDEEGDPMFLRLNIGGAPFILLVDAILRAESTGFLAKFVQLTHP
SRCQVADGFLRHEQAYFFQRSPTAFDAVFQYYSSGVVHRPSEVCPASFLAELEFWRISHQ
HVGSCCADVVPREKEAEKEEEKVDDNTFENLMFGKLRRRMWTFLERPGSSMQAKSFELSS
TLFVLISVMGLSFGTIPDFQVTHYMPPHNETIVLPSGRVKIVEKIEEMRVEHPAFVFTER
ICIAFFTVEYSLRLFAAPRKLRFMMKPLNLVDLLAIVPFYLELILTLCGVDDKKLRDLRW
AFLVVRILRVLRVIRIIKLGRFSSGLQTFGMTLQRSQKQLQMMTIVLLTGVVFFSTMIYF
LEKDEDGTPFTSIPAAYWWCIVTMTTVGYGDAVPATTMGKIIASAAIMCGVLVLALPITI
IVDNFIKVAQDEQTAEQQKMEEENRMAVTSMLNGGDDHDYS
>ppa_mrp-3
MSFGPSTILPDPNSQLDYQQSLMLVGIPVGFFWLLYIPLLIQIKINRGANPVLPWTTIMS
MKWLIAVVLLCDKTFLVLLALWERFIEGDTVPQVEFVSPAVQMITLAVIMLAANGCRRSG
IRSSGILFNTWLIMVLVDQTDFFRYIAYLVWYLLVIIQFILHCFSDPLTIFADEAYDDYP
NCPEMKASFLNQQILYWFGSLITKGNNKLLEVDDLFELKPDLTSEEVVKRWYPIWDKELI
KYQKDTEEFKRKAVVQRKKSTREAAMPLLESGGGRGYGGTNNGDAKKAKNKKKSDEPPLP
SLMYCLLATFKWELMTQNSLKFISDLLLFANPIFLDLLISYTEDPTVAWQIGLLYVAGLF
AAAQLKTFLVNEFFMQALVVGSKVQTLLTAIVYEKTLKLSSHARREKTAGEIVNLMAIDV
ERFKMLVPQLQMYWSSPFQISLTLFMLYQKLGWAAFMGVLVMLSLIPLNIVVSKKIKGWQ
MRMMELKDERIKMCNEVLSGIKVIKLYGWEPAMEKTIDDIRNAEMDLIRKSGILRSGLDV
LNVALVTFATYTLSDPKNVLAPQVAFVSLTLFNQLRGPLMMAAELINQTVQAVVSNQRLK
EFLVADELRPEDIDRLEMLDDDDVKVVDAQEGIFSWGDEIPPTLEGINMDATRGQLLAVV
GRVGSAKSSLLSALLGEMRKLRGYIGTRGTVAYMPQQPWIQNATVRDNILMGMDFDSGKY
NEIVEACALRQDFILLADGDRTEIGEKGINLSGGQKSRVALARACYQDRDVYLLDDPLSA
VDAHVARHIFDKVIGPNGILRKKTRLFVTHGLTFLKDTDKVVIMQDGSISHVDKFEVLVE
DESVSHMLKEVEQVNRKEDMTPSTERSDEHDSADDEDGDQFDDSLSAVSRASRKSKLSVV
SRKISQQGKKLSVTGMPLPEPEKEDKGQLIAKEAMATGHVKASVYFDYIRSMGIWSTFIP
FIAFWTLSSIFQMSRAFWVTAWSNDNII
>ppa_mrp-5
MKPEEDRLHEEEEDEVYPRLHSQKRTKAQGSAKVDEAGILSFVTYHWVFEYLWKAFRGRL
SSDEDWQCSIYDASDVNMARMQVLWDAERSAARTANRPPKLIKAVAAFVKTRVYIACAVF
LFCLIFGFIGPTCFVRGLVGFAEEPGEHSNYTYAFFLVFGLLFVEVARVLSYGATWAISY
RTGIRLRGALLGLLYKHALGVKSQKKTPAEIVNMFANDGQRIFDAITFTPLVLIGPLVLV
GGIIYLMIVIGPWALVGILVFFLFDYGLGKTMVRCRNEAIKKTEERMSLMGELLRCIRAI
KMNGWERAFLDRVQVLRHNEKVSLRKAGYAQSMAIASGPVVPVVAAILTFLGVVLSGNDL
LASDKELVGICGPVGAGKTALLTSIIGHMYPCEGEVEVGGSVALVPQVPWIQNATVQENI
LFGQPMNSKKYYKAISASQLTKDLEAMPANELTEIGERGATLSGGQKARVALARALFSTA
EVLLLDDVLSACDAKVADRIFNDAVLGVLRGKTVLMVTNDVNRLSRCDRVLLMEGGRIVV

SGTHSELLTLSDQYSTYCHDASQRYTLEGDSVVVGVSKEVDKPRPDRVASPTDLEFDHLD
ETNPIVDTEKVELVGLDKGETAKGKLVADEEDFGSASVAFDHNMLLKYVRAAGGVLIWSL
LLFAFLFNVVASIFAPFWLSQWLKHGHDEHMETVNGSEMLVSSDSSLSDSPHTSYYASIY
GISLVLLFVSGLLKAMLFVKVSLNAASRLHNNMLNAIVAGSASFFDSTPSGRLLNRFSKD
VDEIDVKMPFTVEVFLQNMLTCIGFLLVIGWVLPHFVLLSIPLFAVFILFVLCFRAGIRL
MKRSENISRSPLFAHVIHSHDQSIRFLDNMKRRLDSNSAAMFVFQSAMRWLAVWLDLLVV
GITFIVALLIVLLTGKISPSDAGMALAFAIQMSGIFQFAVRTQTELEAKMTSVERVAYYT
DNVAKEGSWDTRPGVQIPADWPARGQID
>ppa_mrp-4
MAVPILNESRALRGLDAFCGERFWDPAVLNSPSLPSFSRCLLHTALVWLPCLFTFLVAPI
LTAQIVYERRPHSLPWTRLLLAKLSIVCVLAADAFFLFVLPFYQASCGSKALFTREVPAA
VEFVYPLMLCLSMLLIGIFILACKHAGKVTAGGLFLSALLFVICGLPELYYWIHVGLHPS
KMPLSDVPRYVAFLVWFPCCVALLLLLSWADAPAAERDGYKELGNEKASPEKSSSFLSRQ
TMWWFNTVCRLGIRKPLEVQDLYALNEDDSSAVLVPKWNKLWGKAMEDFEKRRKLSGVRS
RASTRARSDSTDETPLLAGADRDAYGSTGGAAGPATRVQTTAASAAAATQDVYIAPPSII
ACLFVLFRVDIISAMLVKCVSDLLQFANPLVLDSLIRFTEELHRPLWQGVVLALTMFTAS
ELSSLMLNHYYYLMYRVGTRLQSCLTAAIYKKTLHLSNASRREKTVGEMVNLMAIDVDRF
QQIAPQTMQYWSTPLQIALALYFLWRQMGIATMSGMAVMLFMLPCNFLISMAIRKYQVRQ
MRLKDERTKMVNEVLNGIKVIKLYAWEPPMEGVISNLRDRELSLIRKAAALRTFSDMLNS
ASPFLVAFSTFATFLLIDRKNVLTPQIAFVSLTLFNQLRTPMSTVAELISQTVQVIVSNR
RLKEFLVAEELNPASIDSSAVDNDDVITVTDADMHWDREEPRANLSGLNLTVQKCQLITV
VGKVGAGKSSLLHALLGEMERLRGYVGVRGRAAYVPQQPWMQNQTMRQNITFGKKFDEYF
YNRVLDACALFPDLQMLPLGDMTEIGEKGINLSGGQKARISLARAVYQNHDVYLLDDPMS
AVDSHVGAQLFNAVIGPEGMLRNKTRILVTNELSYLRHANLIVVMKDGKIESEGSYAELM
SNGALQQLLEELYLEDSDSEEFDREELPMESPLIDNVKYAIYYDYFKSMGMSLFLLFVAG
MVISTSVSMGRNFWLSDWSNDNQRSVSS
>ppa_unc-119
>ppa_kvs-1
MAYQSDPNIIWEPHPVFGHIETICILWFTFEYILRIAVAPSRIHFLCGIMNIVDLIAIVP
FFLEQALALFGIDIASLSDIKGALLVVRVLRVLRVVRILKLGRYSSGMRTFALTLKSSAR
QLGMMGMVLSTGVVFFSTLLYFVEKDEKDTPFTSIPAAFWWAIVTMTTVGYGDCVPVTIP
GKLIASGAIISGVLVLALPITIIVDNFMKVSGKMVIPSTRKNQHGSGAGVHNHFRSSQLG
NHHHHSHDDYARQHLPENGRPAPRTDLVSGTGDAVLKLNIGGSPFRLKVSSIFLRGDEGR
LVKFAQLDHEKRVAASDAYFMQSDEYYFERSALLFDAVFKYYATGQLHRPLDVCTHEYSQ
ELSFWKIPENHMSPCCWRNMSESMEDLTNDGQKIEPADRSMRHRIHVFCEGDGSLASTIF
SFASISFVLISVIGLVLGSIHELQVPISKNGTIHVRVAENETTSKLE
>ppa_tph-1
MGHMALFADPDFAQFSQEIGLASLGASEEDLSKLATLYFFSIEFGLSCDSHADKLDSRLK
YKVYGAGLLSSAEELRHAIEGSPSIYRFDPDRVVEQECLITTFQSAYFYTRNFEEAQSKL
RYLCLFDV
>ppa_cat-2
MSCVYHARFKLPFTRAKTPKMRALHSIAESSNQLACSLVMGSIDLSASGAAQSQRRRYSL
VHQASFEKSHIMTLKRANTQKKREELENHLEKNKQASISRDLNDEGVDLIFSLEGNGQLL
FSVIIASSAEISDFLPAVIHTLNDNNVTMQHIETRPEKGGRGVDVLVECAVEHKDSAVSA
VAALKKTHASITAVRMYKTRQDPEVPWFPRHVSEIDKCSHCITKYEPTIDPRHPGFGDEV
YIARREELNRLAANFRYGDQLADVQYTEAEHDTWRQVFDKLEHLHVTLACSTYRRNLAML
LEEGVLTRERIPQLRSINAFLERRTGFILRPCSGLLSARDFLASLAFRVFQGSYENTTQY
LRHHTKPHHSPEPDLIHELLGHVPMFADPALAQMSQDIGLMSLGASDEQIERLATVYWFI
VEFGLCREEGQLRAIGAGLLSAFGELQHACSDVPEHREFIPTETALQKYEDDDYQPLYYV
ADGIEEALGKLREYAGKFERPFSLIYDPYTRSVTEVRSTTASIG
>ppa_pah-1
VFKDRDVNLTHIESRPSKTHEGHYEILVECASDADAHKIEEIIQLFKRRASTVFVHDHNT
RAKQNKESIPWYPQKIADIDQFANRILSYGAELDCDHPGFTDEVYRARRKQFADIAFNFR
HGDKIPRIEYTPQEIATWATVWDALTGLYPKYACKEFNHIFPLMQQNCGYSRDNIPQLQD
VSDFLKDCTGFCLRPVAGLLSSRDFLAGLAFRVFHSTQYIRHHSEPKYTPEPDICHELLG
HAPLFADSEFAQFSQEIGLASLGADDATIEKLATLYWFTIEFGICLQNGEKKAYGAGLLS
SFGELEYAVSIKSSPDQKAPEIESFEPAVTSVQKYPITEYQPKYFLADSFESAKNKLKSW
AATISRPFHVRYNPYTQRIETLDKVTSLQKLAAEINSEITTLQDALGKIKTVA
>ppa_zig-1
MVSPPSSPPPFLSMWWVPALLLALFLHAADALISPPQALNASFGRSSFLHPLTIKEPLTL
WCSTDGIKSAKFVHLSKDKKTYEAKVNGNNATLTIDVPTVLHSGDYRCEMETKNGLKTET
TSVYVRGIVHTEHPEEWKQDDPTKPNFTLGDVVLTEGGSFNLTCPIFSHPLPKINWKKGN

TLIELSSRISVIGDRLTITDVNFDDAGEYTCEALNQYTVDKTRTMRPRLSVTRKVNIKSQ
YAWIWPLAVIIATLILLVVIIWACDIRKKKREKRETTYLATDRE
>ppa_amx-1
MLIAYLFSPKHLTTIHEKLLKIGAVKWTEAEERVFQWNIGNTEFSCGVKLDQVSALYWDQ
NESLVQFAGAHALLIEGSSEIIKKLGEGTDIRMNCPVERVEWEEGKKCAVVCKGGKKYSA
DRIIIAVPLAVLQKGTIEFSPPLPKSKTSAIKGLGAGLIEKIAVRFPRRFWANLEKEDGS
LDYFGHVPSEEKRRGLFNMFYDFSSRGNSKNEHFVLMSYVCGDSVEIVNELSDVEVVKMF
CDTLQDMFGDEKIPEPTGHVVTHWGRDPYIGMSYSYVRVGATGDNYDGIADTVAGKLHFA
GECTNRFFPQTMTGAYLTGVREAGKLLENWKKKE
>ppa_sul-3
MCSREPRTARVFWLLTLIQLAAAAKKPNILLIFADDLGYNDLDWRDSRLHTPFLRSLAFH
KNTVQMNNSYVNQLCTPTRSALMTGYYPFRIGTQSGVFLHMEPTGVPVVFPFVSDNLRAL
GYSTYLIGKWHLGYCNKKFLPTERGFDYFYGYYGPQTGYFNHSADQMHRESGKLVHGLDL
FEEKQQGESKPDFTKGGIYSTDLFSGKAIETLAQHNKDDPFFMFLSFQSVHPPLQVPKSY
ESHCTAFKPNTLRRVYCGMLAAMDVAIARVVKALKQRGLYDNTIIIFSSDNGGGVDFGAS
NWPLRGEKDTLWEGGTRTNTFFHSPQFIKNFAVRNE
>ppa_hst-2
MFKENAFNVVHLNITKNRFVMSLSDQSDLVRNITDWRERQPLFIHGHVAFIDFQRFGVPP
PIYINLIREPFERLLSHYYFLRYGDNYRVGLKRARAGNNETFDECISRSGHDCDPTPLPP
IYPHTLFNTNAYKGYYAVPHKLTWLGVTRDQVDGEIGSRQALEQAKRTLIDKYLVVGVSD
RIRDTVAMLEATIPNFFRGALKHFDSLDDNRAHLRNTKKKIPPSAQTLYLVHSTEVYKLE
KEFYDFALSHFDAQFK
>ppa_hst-1
MYCLVTGDSSPTCSMHPSVHIKYLNLPKWNRQLLDKYSQDYKVPIVSFLPGRAQFFSNSK
VRGMNLTIWHNQKALNIRFSPSSIIPYIARPNVILPFPVSDGREWTLFEDSINFESVVVV
EDIEGKRRAAVIRDKGSVDGVEKIIFGHNLTEWIIGMGFIDALRWSTKCRCGCHSLDRFI
QIDIDDIFVGARGTRMTSDDVEALIQSQDKLRNRISNFSYLLGYSGAFFLHGDDLEDEGD
RMIIEKAANFMWFPHMWKHNHPQDHNESFLESTMIMNKQFGEMNNLRVPFEYAISPQHGG
VYPVHTALFNAWKKVWGVKVTSTEEYPHFRPWGKRKGFIHRGIEVLPRQTCGLYTHTQLF
HLYPGGIKKLTSLIEGGDLFWTIALNPISIFMTHQQNYAHDRLAPYTFENVISFLECWTR
FRLRWEDPISVADRYFHLFPTEKNPIWGNPCMDERHRSILPPTLNCSNLTLPNLLVVGPQ
KTGTTALSSFLQLHPDVSTNRILENTFEEIQFFSGENYEKGFDWYRQLFNNGSSKIVMDK
SATYWDSTEGPMRAFALIPFAKIVVILHDPILRAYSWYNHLVARNESIVRGLSMEDVLNG
DTPAAKKIRSRCISGGRYAHHLDRWLQWFPPSQFHLIDGSELKENPSEVVTNLARWLQLS
PFDFQSLIKFNPKKGFYCKIVSGGMPKYYCDYCDTFLTHDSPSVRKTHNGGRKHKENVRM
YYQKWMEDQAQKLVDATAKAFAAGRGRGMGPPPGVIPPGMMGGMRPPMGMPPMGRGMPPF
GGPPMGGMPPPYMMNRPPMGPGGHRPPMSGLPPMPIDALEFAVEKLEPFDKDKRAYWIKR
LIEGLRKENFNGTVVDLDTLRNVTAAVSTSRNDNIPILNVFDVTSVPRWDIAPDGELIMH
EERKENGLEVDNATMALRQRLMIVQTRISRT
>ppa_hst-3.1
MARCGRVAVIASGLLGSLALIALIEIFSFELSTESGITSTQPLTMARRAMSSLSDAKILP
SRHFPSALLIGVRKGGTRALLDALALHPQIKAARREVHYFDDEMNYGKGEEWYLQQMPFS
RNEITIEKTPAYFTNPLVPERVYRMNPAMKIILIIREPVTRAISDFTQVYYNRLELNKSL
PVFSELAFTREGQIEMSYKPVRNSLYALHLKQWLKIFPLNQILVLDGDIFAKDPLSQLRL
AEDFLGLPHAIRDSQLIFNPSKGFHCFRKTSSSKNMSFRYRVLAIRSIV
>ppa_hst-3.2
MPTSGEGDITIEKSPAYFISKTAPARIKEFNPNMKLIVVLRDPVTRAISDYTQASSKNRR
LGQMPSFERMAVGECAPWSRANCSVKTRGVNGGWGAVRIGVYHKYLERWLKEFPLAQFHF
VDGQKLIDNPADEVKKVEEFLRLKSVVSPADFEVDSVKKFPCIKNPDGTHHCLGKTKGRP
HPKVDEPVLKRLKRFYEPENDKLYSLIHQNFRWDPNLRYRCPAPTAQSSNLT
